# Methylomes of renal cell lines and tumors or metastases differ significantly with impact on pharmacogenes

**DOI:** 10.1038/srep29930

**Published:** 2016-07-20

**Authors:** Stefan Winter, Pascale Fisel, Florian Büttner, Steffen Rausch, Debora D’Amico, Jörg Hennenlotter, Stephan Kruck, Anne T. Nies, Arnulf Stenzl, Kerstin Junker, Marcus Scharpf, Ute Hofmann, Heiko van der Kuip, Falko Fend, German Ott, Abbas Agaimy, Arndt Hartmann, Jens Bedke, Matthias Schwab, Elke Schaeffeler

**Affiliations:** 1 Dr. Margarete Fischer-Bosch Institute of Clinical Pharmacology, Stuttgart, Germany and University of Tuebingen, Auerbachstr. 112, 70376 Stuttgart, Germany; 2 German Cancer Consortium (DKTK) and German Cancer Research Center (DKFZ), Heidelberg, Germany; 3 Department of Urology, University Hospital Tuebingen, Hoppe-Seyler-Str. 3, 72076 Tuebingen, Germany; 4 Department of Urology and Pediatric Urology, Saarland University Medical Center and Saarland University Faculty of Medicine, Kirrberger Straße, 66421 Homburg/Saar, Germany; 5 Institute of Pathology and Neuropathology, University Hospital Tuebingen, Liebermeisterstr. 8, 72076 Tuebingen, Germany; 6 Department of Clinical Pathology, Robert-Bosch-Krankenhaus, Auerbachstr. 110, 70376 Stuttgart, Germany; 7 Institute of Pathology, University Erlangen-Nürnberg, Krankenhausstr. 8–10, 91054 Erlangen, Germany; 8 Department of Clinical Pharmacology, University Hospital Tuebingen, Auf der Morgenstelle 8, 72076 Tuebingen, Germany

## Abstract

Current therapies for metastatic clear cell renal cell carcinoma (ccRCC) show limited efficacy. Drug efficacy, typically investigated in preclinical cell line models during drug development, is influenced by pharmacogenes involved in targeting and disposition of drugs. Here we show through genome-wide DNA methylation profiling, that methylation patterns are concordant between primary ccRCC and macro-metastases irrespective of metastatic sites (r_s_ ≥ 0.92). However, 195,038 (41%) of all investigated CpG sites, including sites within pharmacogenes, were differentially methylated (adjusted P < 0.05) in five established RCC cell lines compared to primary tumors, resulting in altered transcriptional expression. Exemplarily, gene-specific analyses of DNA methylation, mRNA and protein expression demonstrate lack of expression of the clinically important drug transporter OCT2 (encoded by *SLC22A2*) in cell lines due to hypermethylation compared to tumors or metastases. Our findings provide evidence that RCC cell lines are of limited benefit for prediction of drug effects due to epigenetic alterations. Similar epigenetic landscape of ccRCC-metastases and tumors opens new avenue for future therapeutic strategies.

Renal cell carcinoma (RCC) affects approximately 60,000 new patients annually in the United States and 84,400 in the European Union. Histologically, three major subtypes of RCC exist, namely clear cell RCC (ccRCC), chromophobe RCC, and papillary RCC. Among those, ccRCC is the most frequent subtype, accounting for approximately 80% of all RCCs^1^,^2^,^3^. Currently used first line treatment strategy for ccRCC is partial or radical nephrectomy, but patients are at risk of recurrence and development of metastases in different organs after surgery. Thus, several prognostic scores, including DNA methylation-based risk scores, have been developed to identify patients at risk of worse outcome^4^,^5^,^6^. Despite implementation of targeted therapies, using mTOR inhibitors or the multitarget tyrosine kinase inhibitors sunitinib or pazopanib, the survival rate for metastatic ccRCC is only about 20% after 5-year follow-up^1^,^2^,^3^.

Generally, ccRCC is mainly considered as a metabolic disease^7^,^8^ with consequences on treatment strategies. However, the efficacy and toxicity of therapeutic strategies will strongly depend not only on the expression of the drug targets (e.g. VEGF receptor) in primary tumors or their metastases, but also on pharmacogenes involved in drug absorption, distribution, metabolism and excretion (ADME). For instance, genetic polymorphisms in ADME genes like the efflux transporter ABCB1 are associated with survival and toxicity in patients with metastatic RCC treated with sunitinib^9^,^10^,^11^.

The importance of pharmacogenes for the drug discovery process is increasingly recognized, since a substantial proportion of drugs are withdrawn during preclinical stage or during clinical studies not only due to toxicity, but also due to lack of efficacy related to ADME processes like poor drug absorption in target cells^12^,^13^.

In the initial stages of the drug discovery process of novel drugs, cell lines or cell line derived xenograft mouse models play an important role^14^. For instance, the National Cancer Institute introduced an *in vitro* screening platform based on 60 human tumor cell lines (NCI60), including kidney cancers and a recent report demonstrated that use of a large cell-line collection may improve preclinical stratification for anticancer agents^15^. Cell based model systems also served to study molecular mechanisms of drug effects or multidrug resistance. The investigation of pharmacogenes, e.g. using preclinical cell models, is also recommended by the FDA, and for instance preclinical study of membrane drug transporters is part of the submission and approval process of new drugs^16^,^17^,^18^.

Based on cell line experiments, the benefit of DNA demethylating agents as novel therapeutic strategy in RCC has been proposed^19^. This is of importance since epigenetic regulation through DNA methylation is crucial for cancer progression and metastases. Regarding cell line models, there is increasing evidence from several tumor entities other than renal tumors that cell lines might show different DNA methylation or expression patterns compared to primary tumors with consequences on their use as model systems^20^,^21^. For instance, Nestor *et al*. demonstrated that rapid epigenetic re-programming occurred in cell culture of CD4+ T-cells^22^. However, whether pharmacogenes, particularly involved in the absorption, distribution, and metabolism of drugs, display aberrant methylation patterns in RCC cell lines compared to primary tumors or metastases has not been comprehensively investigated, but will be essential for their value as model systems. In general, epigenetic regulation is of importance for the transcriptional expression of ADME genes^23^,^24^ and we could previously demonstrate that drug transporters involved in ADME processes are regulated through DNA methylation, e.g. in primary RCC or hepatocellular carcinoma^25^,^26^.

Moreover, in contrast to other tumor entities^27^,^28^,^29^, it is also unclear whether global DNA methylation patterns and specifically those of pharmacogenes are shared between metastases and primary renal tumors with consequences on gene expression. The identification of particularly metastasis-associated markers may serve as novel targets for therapy in the metastatic setting.

Thus, the aim of our study is to comprehensively investigate genome-wide DNA methylation patterns in ccRCC and distant metastases in different organs to identify epigenetic regulation of pharmacogenes with potential as drug targets or for prediction of drug response. DNA methylation and gene expression profiles are compared to those of RCC cell lines in order to elucidate the predictive value of the established RCC cell lines for the study of pharmacogene-related processes. Validation experiments including gene expression on RNA and protein level corroborate major differences for epigenetic regulation between RCC cell lines and primary renal tumors, while ccRCCs and metastases show similar patterns.

## Results

### Genome-wide DNA methylation in primary ccRCC and metastases

**Figure 1:**
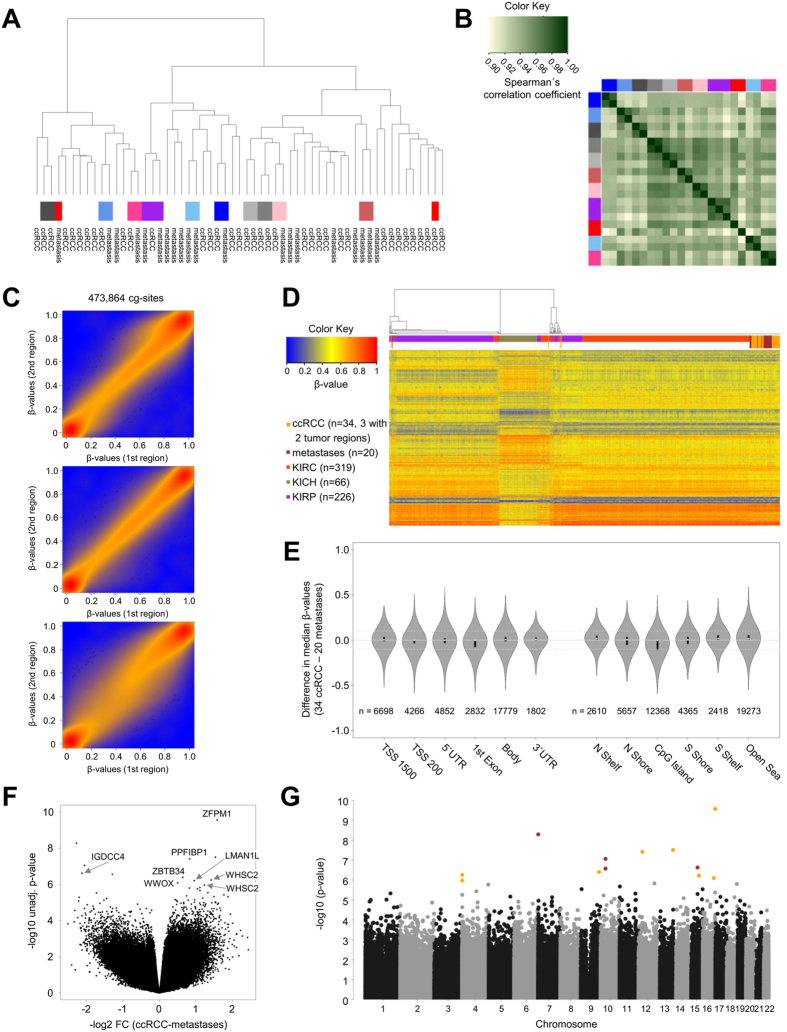
DNA methylome in metastases and primary ccRCC. (**A**) Hierarchical cluster analysis of ccRCC (n = 34) and metastases (n = 20) samples shows similarity between tumor and metastases samples, as well as similarities between samples derived from the same patients (samples from the same patient are marked by identical colors). (**B**) Heatmap showing Spearman’s correlation coefficients for patients (n = 11) for which multiple samples were available. Samples from the same patient are marked as in Fig. 1A by identical colors in the vertical and horizontal side bar. Calculation of correlation coefficients is based on batch-corrected β-values of 473,864 CpG sites (including cross-reactive probes and probes that are impaired by the presence of SNPs). Correlation coefficients are color-coded as indicated. (**C**) Scatter plots with smoothed densities color representation for three different ccRCC tumor tissues, each showing DNA methylation data (β-values) of two regions per tumor. (**D**) Heatmap and cluster dendrogram of primary ccRCC (n = 34), metastases of ccRCC (n = 20), and the three TCGA RCC subtypes (Illumina 450K data on primary tumors: KIRC (ccRCC, n = 319), KICH (chromophobe RCC, n = 66), and KIRP (papillary RCC, n = 226)). The analysis is based on 41,322 CpG sites differentially methylated between the three RCC subtypes. (**E**) Violin plots depicting differences between median β-values of ccRCCs (n = 34) and metastases (n = 20) across various genomic regions. Here, all 46,691 CpG sites with an unadjusted *P*-value ≤ 0.05 between ccRCC and metastases in multivariate analysis were considered. Black bars represent 25% and 75% quantiles; white dots mark the medians, and the gray shape represents the density. (**F**) Volcano plot showing results of multivariate linear mixed model analysis for individual CpG sites, comparing metastases (n = 20) and primary ccRCC tumors (n = 34). (**G**) Manhattan plot showing results of multivariate linear mixed model analysis for individual CpG sites, comparing ccRCC (n = 34) and metastases (n = 20) samples. Analyses are based on 473,864 CpG sites (including cross-reactive probes and probes that are impaired by the presence of SNPs). Significantly different CpG sites that are hypomethylated in metastasis compared to primary ccRCC are marked in orange and hypermethylated ones are marked in brown.

Genome-wide DNA methylation analyses of tissue samples of our cohort (Table 1) were performed using the Illumina HumanMethylation 450 BeadChip. In total, 20 metastases in different organs (Table 1) and 34 primary ccRCC tumors were investigated, including tumor and metastasis tissue that were derived from the same patient (n = 6) as well as metastases occurring in different organs of the same patients (n = 3). Prior to all our analyses, methylation sites located on the X- and Y-chromosome as well as control probes were excluded, resulting in 473,864 CpG sites. As shown in Fig. 1A, all metastases clustered with the primary ccRCC tumor samples, indicating that global DNA methylation profiles in metastases of ccRCC show a high similarity to DNA methylation patterns observed in primary ccRCC samples. Of note, methylation patterns of samples derived from the same patient showed not only a high correlation (r_S_ > 0.96) (Fig. 1B), but also small absolute differences in DNA methylation levels (Supplementary Fig. S1A,B; mean absolute differences in β-values  < 0.060), except for one case (marked in red) which revealed a slight deviation in β-values (mean absolute differences in β-values  = 0.092) and lower correlation (r_S_ = 0.92) (Fig. 1A,B). Moreover, the local recurrence samples clustered with the primary tumor and distant metastasis samples (Supplementary Fig. S1C). In addition, we analyzed intra-tumor heterogeneity of DNA methylation in three ccRCC tumors from which genome-wide DNA methylation data were obtained from two regions of each tumor. As shown in Fig. 1A, samples derived from the same tumor (marked in identical grey shades) clustered together and DNA methylation patterns of these samples derived from the same tumor were not only significantly correlated (r_S_ > 0.97; Fig. 1B,C), but also showed similar methylation levels (mean absolute differences in β-values 0.026, 0.038, and 0.047, respectively; Fig. S1B). Of note, their correlation coefficients and differences in methylation levels were comparable to those of technical replicates (r_S_ = 0.99 and mean absolute differences in β-values  = 0.020; Supplementary Fig. S2).

### Genome-wide DNA methylation in RCC subtypes and metastases

We next compared the DNA methylation data of our cohort to those of three RCC subtypes collected by TCGA: ccRCC (KIRC; n = 319), chromophobe RCC (KICH; n = 66), and papillary RCC (KIRP; n = 226). For this purpose, cluster analysis was performed based on 41,322 CpG sites which differed significantly and relevantly (Benjamini-Hochberg –adjusted [BH-adj.] *P-*value ≤ 0.05, absolute difference in median β-value ≥ 0.20) between the three RCC subtypes of TCGA. As shown in Fig. 1D, all RCC samples were clustered into three subgroups separating mainly ccRCC, chromophobe and papillary RCC subtypes. Interestingly, 15 ccRCC samples of the KIRC TCGA cohort revealed DNA methylation profiles similar to the majority of chromophobe RCC signatures. Moreover, ten ccRCC samples of the KIRC TCGA cohort displayed a papillary-like pattern. Re-evaluation of the histological diagnosis of these cases by two independent pathologists with expertise in renal tumor pathology was performed using the publicly available TCGA diagnostic and histological slide images. Results from this re-evaluation indicated that the 15 chromophobe-like ccRCC cases include chromophobe RCC, oncocytoma and other rare variants of RCC and the 10 papillary-like ccRCC cases also include non-ccRCC samples, thereby confirming our results from the cluster analysis (Supplementary Table S1). Almost all metastases samples of our cohort, irrespective of the affected organ, as well as the primary ccRCC samples of our cohort, clustered with the ccRCC samples (KIRC) of the TCGA cohort. Only one metastasis sample, derived from the pancreas, showed a papillary-like pattern and one of our primary ccRCC samples displayed a chromophobe-like signature. Additionally, one tumor sample was not consistently clustered within the ccRCC cohort, indicating a clear cell RCC phenotype with papillary features.

### Concordant DNA methylation in primary ccRCC and metastases

Further analyses to identify individual CpG sites or gene regions that are differentially methylated between metastases and primary tumor tissue of our cohort were performed using linear mixed model analyses. Here, we corrected for age, sex, array batch and also considered that samples were in part derived from the same patient (five patients with paired RCC and metastasis, one patient had one RCC and two metastases, and two patients with two metastases each; regarding the three ccRCCs for which two regions of each tumor were measured, DNA methylation levels were averaged prior to all linear mixed model analyses; see Supplementary Methods). We first investigated whether alterations occur depending on the genomic location of CpG sites. As depicted in the Violin plots (Fig. 1E), no considerable differences in promoter regions, CpG islands or in the gene-bodies were found. Notably, only a few significantly differentially methylated CpG sites were identified after adjustment for multiple testing (Fig. 1F,G; Supplementary Table S2). Interestingly, moderately increased methylation levels in metastases compared to primary tumors were observed for a CpG site in the *IGDCC4* (immunoglobulin superfamily, DCC subclass, member 4) gene region (Supplementary Fig. S3). Of note, no significantly differentially methylated regions (DMR) were observed when at least three CpG sites with BH-adj. *P* ≤ 0.05 per region were required. Significant DMR, using a relaxed false discovery rate (FDR) threshold of 15% for at least two CpG sites per region, are shown in Supplementary Table S3 and Supplementary Fig. S4. Here, DNA methylation in *WHSC2* was reduced in metastases compared to ccRCC tumors, whereas *GALR1* methylation was increased.

Next, DNA methylation profiles were compared between metastases occurring in lymph nodes and those in other organs to investigate whether DNA methylation is influenced by the organ in which the ccRCC metastasis occurred. No differentially methylated gene regions or CpG sites could be identified after adjustment for multiple testing (Supplementary Fig. S5A). Additionally, DNA methylation patterns between synchronous and metachronous metastasis were compared and no statistically significant differences were found after adjustment for multiple testing (Supplementary Fig. S5B), even when excluding the local recurrence samples (data not shown). The same held true when CpG sites that are influenced by SNPs and probes with potential cross-hybridization were excluded (data not shown). Additionally, restriction of analyses to patients without metastatic ccRCC (M0 and N0), or comparing metastases with either primary RCCs for which no metastasis occurred during follow-up, or primary RCCs for which one or more metastases occurred during follow-up did not reveal additional significantly different CpG sites (Supplementary Fig. S5C–F).

Exemplarily, DNA methylation patterns of candidate genes *TINAGL1, ESYT3, ITGA5, FKBP10*^30^, and CYTIP^31^ recently identified to be involved in epithelial-to mesenchymal transition (EMT) are depicted in Supplementary Fig. S6A,B. None of these regions were significantly differentially methylated in metastasis samples compared to primary tumor samples of our cohort after adjustment for multiple testing. Moreover, no significant differences between methylation levels in tumor and metastases were observed in the *VHL* promoter region (Supplementary Fig. S6C), which is known to be differentially methylated in about 7%^7^ of ccRCC compared to non-tumor tissue.

### Genome-wide alterations of DNA methylation in RCC cell lines

**Figure 2:**
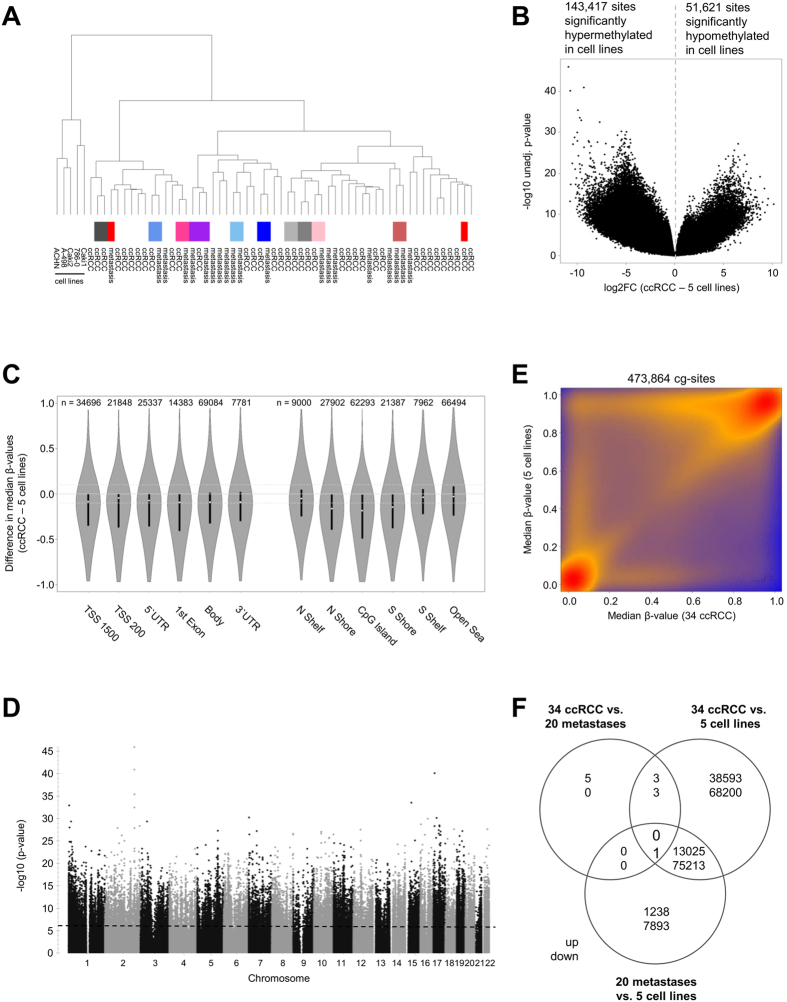
DNA methylome in RCC cell lines. (**A**) Hierarchical cluster analysis of five RCC cell lines, ccRCC (n = 34) and metastases (n = 20) samples (samples from the same patient are marked by identical colors). (**B**) Volcano plot showing results of multivariate linear mixed model analysis for individual CpG sites, comparing RCC cell lines and primary tumors. (**C**) Violin plots depicting differences between median β-values of ccRCCs (n = 34) and RCC cell lines (n = 5) across various genomic regions. Here, 195,038 CpG sites significantly differing between ccRCC and RCC cell lines in multivariate analysis were considered. (**D**) Manhattan plot showing results of linear mixed model analysis of differentially methylated CpG sites, comparing ccRCC (n = 34) and five RCC cell lines. Analyses are based on 473,864 CpG sites (including cross-reactive probes and probes that are impaired by the presence of SNPs). (**E**) Scatter plots with smoothed densities color representation of median DNA methylation levels (β-values) in primary ccRCC (n = 34) and RCC cell lines (n = 5), indicating hypermethylation of RCC cell lines compared to primary tumors. (**F**) Venn diagram depicting the number of significantly hyper-and hypomethylated CpG sites in pairwise comparisons of primary tumors, metastases and RCC cell lines.

Furthermore, genome-wide DNA methylation profiles of five RCC cell lines (Caki-1, Caki-2, A-498, ACHN, and 786-O) were compared with 20 metastases and 34 primary ccRCC tumors of our cohort. As shown in Fig. 2A, cell lines did not cluster with metastases or tumor samples. Detailed analyses revealed that genome-wide 195,038 individual CpG sites (41.2%) differed significantly between RCC cell lines and primary tumors (Fig. 2B), and 97,370 sites (20.5%) differed between RCC cell lines and metastases samples, respectively. Among those CpG sites, 41,682 and 8,194 CpG sites with an adjusted *P* < 1E-05 were found comparing cell lines and primary ccRCC (Fig. 2B) or metastases, respectively. Further analyses considering genomic locations showed that differences occurred in all investigated regions. However, predominantly hypermethylation in CpG islands and related shores was observed in cell lines compared to primary tumors (Fig. 2C). Furthermore, CpG sites throughout the genome were altered in cell lines compared to primary ccRCC (Fig. 2D). 73.5% of CpG sites were significantly hypermethylated in the five investigated cell lines compared to primary ccRCC (Fig. 2E,F). The same trend was observed for the comparison of cell lines and metastases (Fig. 2F).

**Figure 3:**
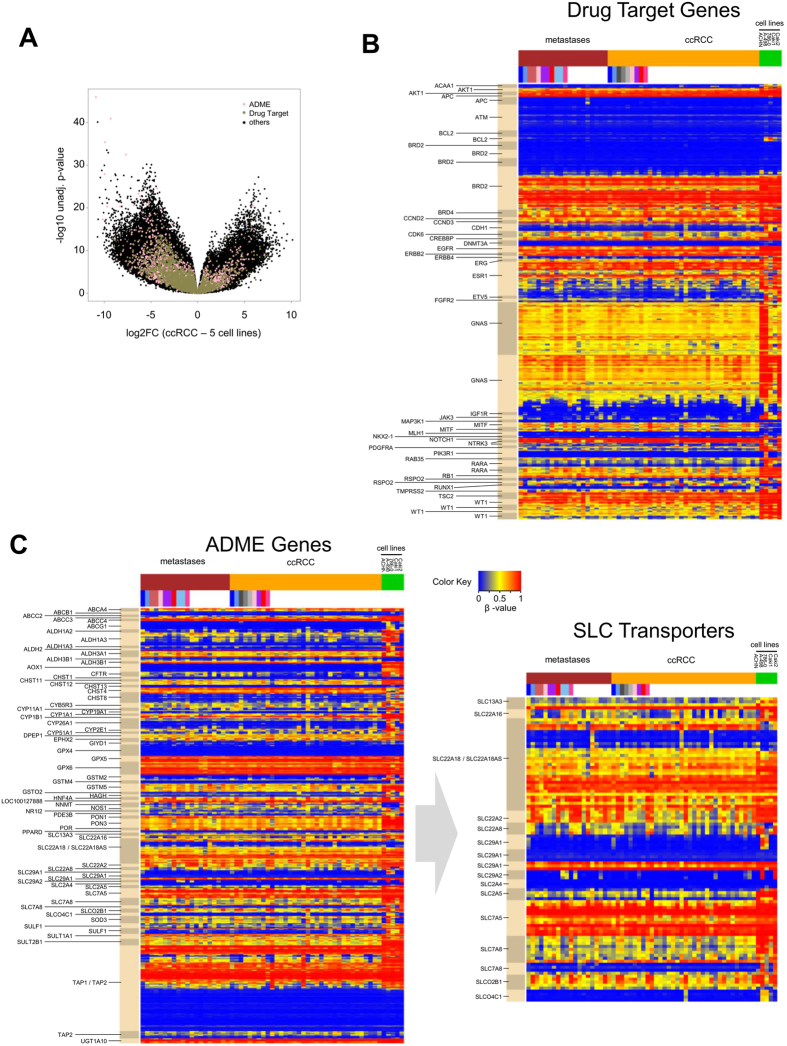
DNA methylation of pharmacogenes. (**A**) Volcano plot showing differentially methylated CpG sites in ADME and drug target genes between RCC cell lines and primary tumors. (**B**,**C)** Cluster analyses showing significantly differentially methylated gene regions (DMR) within (**B**) drug target genes or (**C**) ADME genes and particularly membrane transport proteins of the solute carrier (SLC) family.

Since RCC cell lines are frequently used to study the effects of drugs and for identification of drug targets, we asked whether pharmacogenes particularly involved in ADME processes like drug uptake or drug targeting are differentially methylated in RCC cell lines compared to primary RCC. A list of ADME related genes, which is based on the PharmaADME initiative (www.pharmaadme.org), as well as drug targets (based on www.broadinstitute.org/cancer/cga/target) was assembled (see Supplementary Table S4). The list contains 298 genes involved in e.g., drug metabolism or drug transport and 135 drug target genes, such as *EGFR* or *BRAF*. In summary, 1981 (41.7%) CpG sites within ADME genes and 1688 (39.3%) individual CpG sites within target gene regions were significantly (adjusted *P* < 0.05) differentially methylated between cell lines and primary ccRCC (see Volcano plot Fig. 3A). Moreover, 399 (8.4%) of those CpG sites within ADME genes and 366 (8.5%) individual CpG sites within target gene regions were differentially methylated between cell lines and primary ccRCC with an adjusted *P* < 1E-05 (see Volcano plot Fig. 3A). Most of these significant sites in ADME (78.0%) and drug target genes (82.5%) were hypermethylated in cell lines compared to primary ccRCC tumors, which is comparable to the methylation alterations observed for other genes.

In addition to individual CpG sites, also differentially methylated gene regions (DMR) were identified within drug target genes (n = 53; e.g. *JAK3*; Fig. 3B) or ADME genes (n = 74; Fig. 3C) with *P* < 0.006. We next focused on drug transporters which play an important role in the uptake and efflux of drugs in cancer cells, thereby contributing to drug resistance and non-response. Notably, several important uptake transport proteins are hypermethylated in RCC cell lines (e.g. *SLC22A2, SLC22A8*; Fig. 3C, right panel). In contrast, DNA methylation levels of ADME genes or drug targets were similar between metastases and primary ccRCC samples, as shown in Fig. 3B,C.

### Epigenetic regulation of the expression of pharmacogenes

**Figure 4:**
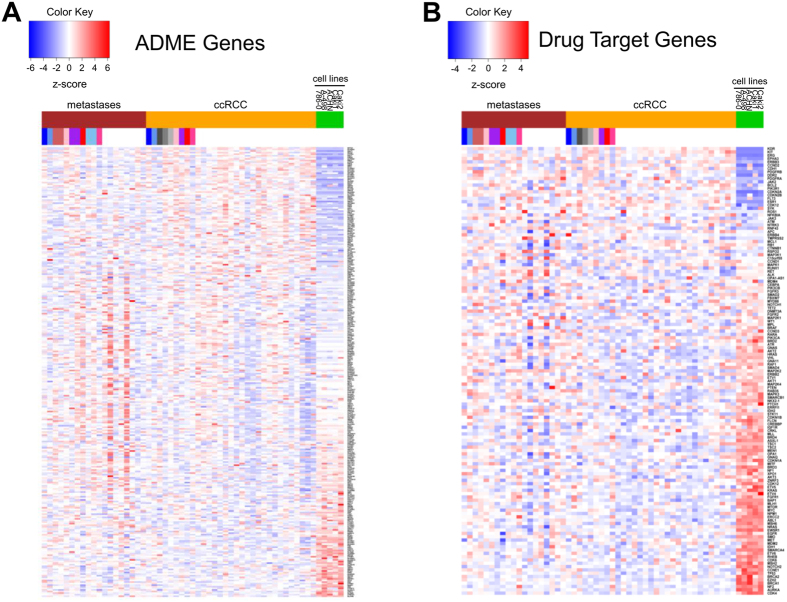
Gene expression of pharmacogenes in primary tumors, metastases and RCC cell lines. Heatmaps showing significantly differentially expressed ADME genes (**A**) or drug target genes (**B**).

Since aberrant methylation of pharmacogenes and drug targets might result in altered gene expression of the respective genes, we next investigated their transcriptional expression in cell lines as well as primary ccRCC and metastases samples using Affymetrix Human Transcriptome 2.0 microarrays. Gene expression levels of 134 ADME and 93 drug target genes were significantly altered in cell lines compared to primary ccRCC tumors (Fig. 4A,B). Notably, about 7% of ADME genes (e.g., organic cation transporter OCT2) normally expressed in ccRCC tumors and metastases were expressed at very low levels or even not expressed at all in RCC cell lines. The same was true for drug target genes (e.g., type III receptor tyrosine kinase *KDR*, known as *VEGFR*). We further performed correlation analyses of DNA methylation levels and gene expression of ADME genes and drug targets. Therefore, only CpG sites significantly and relevantly (absolute difference in β-values > 0.1) differentially methylated as well as genes significantly and relevantly (absolute log2 fold change > 1, mean log2 signal intensity > 6) differentially expressed between primary tumors and cell lines were considered. For each gene, Spearman’s correlation coefficients were calculated between the mRNA expression and the DNA methylation levels of the CpG sites within the gene. As shown in Table 2, significant correlations between DNA methylation and gene expression were detected for 18 ADME as well as 8 drug target genes (results for each individual CpG site are given in Supplementary Table S5). Focusing on DNA methylation in promoter regions (TSS gene regions), which might directly influence gene expression levels, revealed significant correlations for e. g., the organic cation transporter OCT2/SLC22A2 and the drug target KDR (Supplementary Table S5).

**Figure 5:**
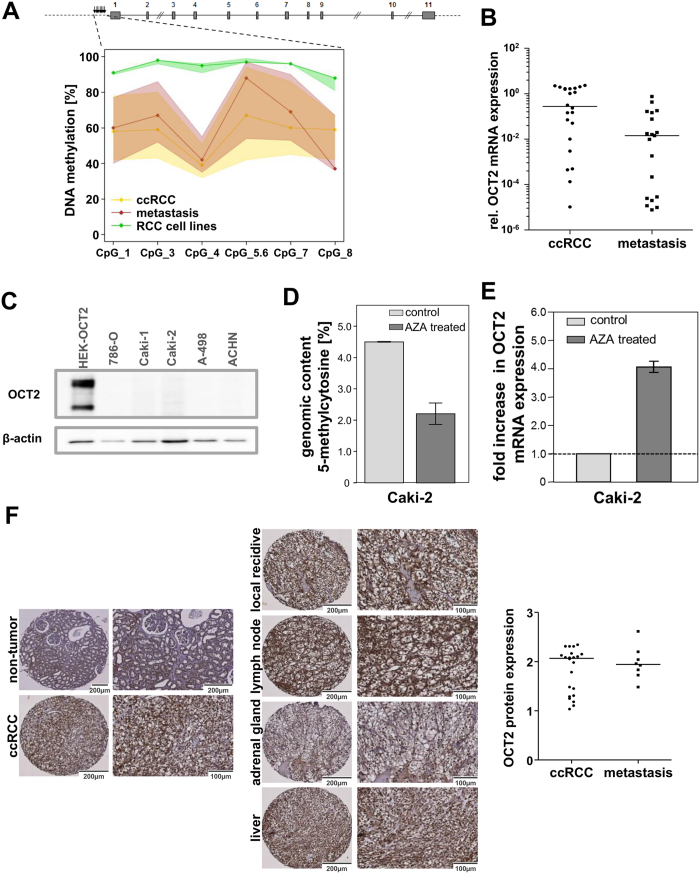
DNA methylation, mRNA and protein expression of SLC22A2/OCT2 in ccRCC, metastases, non-tumor kidney tissue, and RCC cell lines. (**A**) DNA methylation at the *SLC22A2* promoter in metastases, ccRCCs and five RCC cell lines was quantified using MALDI-TOF MS. No significant differences between metastases and ccRCCs were observed, but DNA methylation in all five RCC cell lines is significantly increased compared to tumor and metastases tissue. (**B**) SLC22A2/OCT2 mRNA expression in tumor and metastases tissue samples. mRNA expression was determined by quantitative real-time PCR and normalized to β-actin expression. (**C**) Protein expression of OCT2 in five RCC cell lines investigated through Western blotting revealed that OCT2 is not expressed in RCC cell lines on protein level. OCT2 transfected HEK-cells served as positive controls. β-actin expression was used as loading control. (**D**) Effect of treatment of Caki-2 cells with 5-Aza-2´-deoxycytidine (AZA) on global DNA methylation. Cells were either untreated or treated with 1 μM AZA and the amount of 5-methylcytosine was quantified using LC-MS-MS to verify the effect of AZA treatment on global DNA methylation. Results represent mean of 2 experiments ± SE. (**E**) Effect of AZA treatment on mRNA expression. Cells were cultured with 1 μM AZA and mRNA levels (normalized to β-actin) were determined using TaqMan technology. Fold increase in expression compared to untreated cells was calculated. (**F**) Immunohistochemical staining of OCT2 exemplarily in normal kidney tissue, ccRCC tissue samples and metastases of ccRCC. Protein expression was investigated in TMAs by semiquantitative immunohistochemistry (right panel), indicating that OCT2 is expressed both in ccRCC and metastases.

The organic cation transporter 2 (encoded by the *SLC22A2* gene) is well characterized as uptake drug transporter for platinum drugs as well as other anti-cancer agents and the current FDA Guidance^32^ on “Drug Interaction Studies” recommends OCT2 as an important ADME target to be considered in the preclinical/clinical drug development process. Therefore, we further investigated its DNA methylation and expression on mRNA and protein level. Since the Illumina HumanMethylation 450 BeadChip does not cover every single CpG site in the human genome, we first validated DNA methylation data through more comprehensive MALDI-TOF mass spectrometry analyses. The MALDI-TOF MS assay is located in the *SLC22A2* promoter region which was previously identified to be important for regulation of OCT2/SLC22A2 expression^24^,^33^. As shown in Fig. 5A, DNA methylation levels were significantly increased in RCC cell lines. These results were consistent in three replicates. In addition, no significant differences between DNA methylation in metastasis and tumor tissue were observed (Fig. 5A). Next, we analysed SLC22A2/OCT2 expression on mRNA and protein level. The mRNA levels were similar between primary tumor and metastasis tissue (Fig. 5B), but in RCC cell lines the OCT2 expression was below the limit of quantification. Investigation of OCT2 protein expression through Western blotting revealed that OCT2 protein was not detectable in these cell lines (Fig. 5C). To further assess a causal association between DNA methylation and expression, we examined the effect of AZA (5-Aza-2′-deoxycytidine, decitabine), which is a well-established DNA methylation inhibitor, on OCT2 expression in Caki-2 cells. To verify the effect of AZA treatment, global DNA methylation status was determined using an established LC-MS-MS method^34^. The proportion of 5-methylcytosine residues in genomic DNA of Caki-2 cells decreased from 4.50% to 2.20% after treatment with 1 μM AZA, clearly demonstrating the demethylating effect (Fig. 5D). Subsequently, mRNA expression was analyzed using real-time PCR technology. As shown in Fig. 5E, treatment of Caki-2 cells led to an increase of expression of OCT2. Since especially SLC transporters (Fig. 3C) displayed alterations in DNA methylation, we next assessed the expression of 55 SLC transporters in Caki-2 cells treated with decitabine using real-time PCR technology. Quantification of their mRNA expression levels indicated that several transporters are upregulated on mRNA level through demethylation (see Supplementary Fig. S7). Since organic cation transporters mediate cell sensitivity to platinum drugs like cisplatin^35^, we tested the effects of 5-Aza-2′-deoxycytidine (decitabine) pretreatment on cisplatin sensitivity in Caki-2 cells. The percentage of dead cells was quantified by FACS analysis using Annexin V staining^36^. As shown in Supplementary Fig. S8, the combination of both drugs showed significantly greater induction of apoptosis than either drug alone.

In addition, OCT2 protein expression in metastatic and primary tumor tissue was investigated by immunohistochemical staining of tissue microarrays. Representative staining of tissue cores is shown in Fig. 5F. In all metastasis samples, irrespective of the involved organ, a strong membranous staining of OCT2 was observed (Fig. 5F, left panel). Semiquantitative analyses of protein expression levels indicated that expression levels in metastases were within the range of tumor tissue (Fig. 5F, right panel). Thus, macro-metastases of ccRCC not only retain their *SLC22A2* DNA methylation profile, but as a consequence also display the same high OCT2 expression as primary ccRCC tumors, irrespective of the affected organ. In RCC cell lines, OCT2 protein expression is downregulated due to hypermethylation in the *SLC22A2* promoter region compared to primary tumors or metastases.

## Discussion

Increasing evidence suggests that epigenetic mechanisms play an important role in the dynamic process of metastasis. Therefore, detailed knowledge not only of the epigenetic alterations present in primary tumor tissue, but also in metastatic tissue is of great interest especially for the development of novel treatment strategies for patients with metastatic disease. However, the efficacy of all therapeutic strategies will depend on the uptake, metabolism or excretion of the drugs in the tumor tissue as well as in metastases. For drug discovery and cancer research, cancer cell lines are still essential and extensively used, despite knowledge about their limitations.

In-depth molecular profiling on primary ccRCC tumors and non-tumor kidney tissue has been performed e.g. by The Cancer Genome Atlas (TCGA). However, corresponding metastases of ccRCC were not studied in the TCGA initiative. Since therapeutic response to anticancer agents might differ between primary tumors and metastases, we performed for the first time a comprehensive analysis of genome-wide DNA methylation in ccRCC metastases including local recurrence and metastasis samples derived from different organs (e.g. lymph node, liver, lung or adrenal gland). Moreover, paired tumor and metastases tissue as well as different metastases of the same patient were investigated.

Cluster analyses including all samples from our cohort as well as samples from TCGA of different RCC entities (clear cell, chromophobe, and papillary RCC) confirmed the ccRCC subtype in all our metastatic tissues, except for one metastasis. Interestingly, some ccRCC samples of the TCGA KIRC cohort did not cluster within the ccRCC cluster. Histopathological re-evaluation confirmed a non-ccRCC morphology and phenotype in most of these samples (Supplementary Table S1). This is in agreement with our previous findings using gene expression levels^4^ and results of the TCGA consortium, that in some cases the tumors were not conventional ccRCCs (see Supplementary Information in ref. 7). Furthermore, the present findings indicate that subtypes of RCC can be identified based on DNA methylation levels, in line with recent findings from Malouf *et al*.^37^.

In general, our data provide novel evidence that DNA methylation profiles are shared between primary ccRCC tumors and corresponding macro-metastases in various distant organs. Moreover, DNA methylation levels of CpG sites in gene regions previously identified to be important in epithelial(-to-)mesenchymal transition (EMT) in renal cell models are not altered in macro-metastasis. For instance, DNA methylation patterns of four candidate gene regions (*TINAGL1, ESYT3, ITGA5, FKBP10*), recently identified to be involved in EMT in a canine kidney cell model and confirmed in breast cancer^30^, were not significantly differentially methylated in metastasis samples compared to primary tumor samples of our cohort. The same result was found for the cytohesin interacting protein CYTIP, previously identified to be important in the process of EMT in ccRCC^31^.

Since we investigated metastases of ccRCC in nine different organs (Table 1), we had the possibility to examine the DNA methylome in its organ microenvironment, which cannot be restored using cell lines or cell line-derived xenografts. Our findings indicate that alterations of DNA methylation in metastasis generally seem not to be majorly impaired by the microenvironment of distant organs. Based on the hypothesis of two recent publications that clusters of cells that escape from the primary tumor have a higher ability to form distant metastasis compared to circulating single cells^38^,^39^, our results indicate that DNA methylation features influenced by the ccRCC microenvironment might be preserved in these cell clusters during the process of macro-metastases formation.

Notably, also DNA methylation and expression patterns of pharmacogenes, such as drug target genes and ADME-related genes, were shared between primary tumors and metastases. Thus, ADME processes, e.g. drug uptake processes and subsequent drug effects are comparable between tumors and metastases. However, all five studied RCC cell lines displayed not only genome-wide hypermethylation compared to primary tumors or metastases, but also altered DNA methylation of drug targets and important ADME genes was observed. Our data indicating that the investigated renal cancer cell lines generally are hypermethylated compared to primary renal tumors, are in line with previous data of other tumor entities (e.g. breast, prostate, colon) demonstrating CpG island hypermethylation in cancer cell lines compared to primary tumors^40^. The effect of cell culture on epigenetic alterations was also investigated for other tumor entities through genome-wide analyses of cell lines and primary tumors by Varley *et al*.^20^, though the underlying mechanisms have not been completely elucidated. Antequera *et al*.^41^ proposed that CpG island hypermethylation in cell culture is associated with the loss of DNA demethylase activity and recently, Nestor *et al*.^22^ provided first evidence that loss of TET-mediated demethylase activity is responsible for cell culture-induced hypermethylation.

In our study, we could further demonstrate that alterations in DNA methylation lead to expression differences in pharmacogenes involved in ADME processes. For instance, important drug transporters (e.g. the organic cation transporter OCT2/SLC22A2) were expressed at high levels in primary tumors and metastases, but seem to be downregulated in any of the investigated RCC cell lines due to hypermethylation. Interestingly, these cell lines differ in their basal phenotypes and genomes, such as the mutation status in the von Hippel-Lindau (VHL) gene. Although DNA methylation analyses through Illumina HumanMethylation 450 BeadChip is highly reliable, not every single CpG site present in the human genome is covered by the array. Moreover, some DNA methylation values are impaired by cross-reactive probes present on the microarray. Thus, additional experiments using independent methods e.g., MALDI-TOF MS or pyrosequencing, might still be necessary not only for validation, but also for fine-mapping of DNA methylation in the promoter region to identify single, relevant CpG sites not covered by the Illumina HumanMethylation 450 BeadChip, as we recently investigated for the monocarboxylate transporter MCT4^25^.

Notably, in the present study the results of genome-wide methylation analyses of *SLC22A2* were validated by gene-specific analyses, using an independent MALDI-TOF MS assay covering the CpG site previously identified to regulate OCT2 expression. Further analyses of the expression data from 533 tumors of the TCGA ccRCC cohort indicated that indeed 88% of all ccRCC tumors showed an OCT2 expression, which was higher than the median gene expression of all genes expressed in ccRCC (Supplementary Fig. S9A). Moreover, based on TCGA data, a significant correlation between DNA methylation and mRNA expression in ccRCC tumors exists (Supplementary Fig. S9B). In addition, immunohistochemical staining of OCT2 in primary ccRCC tumors and metastasis demonstrated that OCT2 was highly expressed in all metastases of ccRCC irrespective of the organ and its expression was found to be quite homogeneous in metastases of ccRCC. In contrast, OCT2 protein was not detectable in any of the five studied RCC cell lines. This finding results in important consequences because OCT2 is known to be involved in the uptake of anticancer agents (e.g. platinum drugs or the survivin inhibitor YM155) and is one of the key transporters that needs to be evaluated in drug development according to the International Transporter Consortium^16^,^42^. Based on our data, DNA methylation (Fig. 3C) and expression of several uptake transporters potentially relevant for novel cancer drugs are altered in RCC cell lines compared to primary tumors and metastases indicating that these cell lines do not sufficiently reflect conditions *in vivo*. Thus, in case of novel drugs developed for treatment of RCC in the future this discrepancy is of major importance for valid interpretation of preclinical data derived from RCC cell line experiments and to avoid misleading conclusions for further steps in the drug development process. Probably treatment of cells with demethylating agents might be promising in order to increase SLC transporter expression in cell lines, corroborated by the fact that exemplarily decitabine pretreatment results in higher sensitivity of cells to cisplatin (Supplementary Fig. S8). Generally, demethylating agents like 5-Azacytidine or decitabine have been approved for therapy of myelodyplastic syndrome, but have also been proposed as therapeutic option for solid tumors. Previously, Ricketts *et al*. provided preclinical evidence that treatment with demethylating agents might be useful for RCC^19^. Since metastases and primary tumors share similar methylation profiles, use of demethylating agents alone or in combination might be promising for treatment of metastatic patients. Combination therapy using decitabine and platinum drugs has previously been reported to be more effective in tumor models^43^, most likely because they exerted their effects through different mechanisms^44^. Recently, even an immune-based mechanism of action of DNA methyltransferase inhibitors has been proposed^45^. Thus, combination of for instance demethylating agents and new immune-checkpoint–inhibitors, such as those blocking programmed death 1 (PD-1), might be a promising option for treatment of ccRCC.

Although we provide the first data on DNA methylation in metastasis of ccRCC, our retrospective study has some limitations. Of course, we cannot exclude that even small differences between metastases and primary tumors in individual CpG sites might be of biological relevance, which were not statistically significant because of lack of statistical power due to the size of our cohort. The observed alterations in gene expression might not only be caused by epigenetic factors, but also by genetic factors known to play an important role in gene regulation. Genetic variants which might additionally alter variability of DNA methylation have not been systematically investigated, although consideration of SNPs present on the Illumina HumanMethylation 450 BeadChip revealed no significant impact of genetic variants (data not shown). As shown in Table 1, some of the patients received treatment before surgery for metastasis; however, statistical analyses including prior treatment indicated no influence (data not shown). Whether prior treatment decreases the heterogeneity of DNA methylation in RCC and metastasis as previously suggested for colorectal cancer, has not been studied. However, our immunohistochemical staining of OCT2 protein in metastases of different organs is rather homogeneous irrespective of prior treatment suggesting limited evidence of intra-tumor heterogeneity at least for OCT2. Moreover, we found a significant correlation of DNA methylation patterns derived from two regions of one tumor. Nevertheless, further studies investigating heterogeneity in more samples of each organ (e.g. lung) are needed.

In addition, further studies need to evaluate whether primary RCC cells derived from tumor or metastases tissue are more appropriate to study pharmacogene-related processes. However, data from primary renal proximal tubule cells demonstrated that the expression of drug transporters is down-regulated indicating also limited applicability of primary cells to study drug transport processes^46^,^47^. Spheroids, as well established three-dimensional (3D) models for drug screening, might represent an interesting alternative to improve preclinical drug response prediction^48^. In this context, patient derived tumor xenografts (PDX) might be used as well to study not only cancer biology, but also drug related processes^49^. There are first data regarding PDX models for RCC and especially for the ccRCC subtype^50^,^51^, indicating that PDX models preserve gene expression profiles of the primary tumors^52^. Only recently, Kim *et al*.^53^ provided evidence that PDX models (including models derived from a corresponding metastasis and single cells derived from PDX-tumor and metastasis) might be promising to design personalized therapeutic strategies for ccRCC. Whether DNA methylation profiles of pharmacogenes are maintained in these models needs to be evaluated, but such models offer great potential to study drug related processes.

Taken together, the present study not only provides new insight into the epigenetic landscape of ccRCC derived metastases with impact on therapy or diagnosis, but additionally elucidates limited benefit of RCC cell lines for investigating pharmacogene-related processes.

## Materials and Methods

### Study cohort

Metastases (n = 20) as well as primary tumor samples (n = 34) of ccRCC patients were obtained from the Departments of Urology and Pathology (University Hospital Tuebingen, Germany), as well as the Department of Pathology (Robert Bosch Krankenhaus, Stuttgart, Germany) (see Supplementary Methods). Patients’ characteristics, clinicopathological features, survival data as well as details about metastases are given in Table 1. Informed written consent was provided by each subject and use of the tissue was approved by the ethics committee of the University of Tuebingen, Germany. All methods were carried out in accordance with the approved guidelines.

Additionally, publicly available DNA methylation data of The Cancer Genome Atlas (TCGA) from three independent cohorts of RCC patients (n = 611) including primary ccRCC (KIRC), chromophobe (KICH) and papillary RCC (KIRP) were analyzed. Details about analyses of TCGA data is given in Supplementary Methods.

### RCC cell lines

A-498, 786-O, Caki-1, and ACHN cell lines (purchased from CLS Cell Lines Service, Eppelheim, Germany), as well as the Caki-2 cell line (purchased from Sigma-Aldrich, Taufkirchen, Germany) were cultivated as previously described^25^. Cell lines were routinely tested for mycoplasma infection using a PCR-based test (Venor^®^GeM Classic, Minerva Biolabs GmbH, Berlin, Germany) and authentication of cell lines was performed using the PowerPlex^®^ 21 System (Promega, Madison, USA) according to the manufacturer’s protocol.

### Analyses of DNA methylation and gene expression

Genome-wide DNA methylation profiles of 20 metastases of ccRCC in different organs (see Table 1) and 34 primary ccRCC tumors of our cohort, as well as five RCC cell lines were generated using the Illumina Infinium HumanMethylation 450 BeadChip. Genomic DNA was purified from fresh-frozen ccRCC and metastasis tissue, as well as cell lines using standard procedures (QIAamp DNA Mini Kit). For three tumor samples, DNA was isolated from two regions of the tumor tissue and independently processed. Bisulfite conversion and further analyses using Illumina HumanMethylation 450 BeadChip were performed by Service XS (Service XS, Leiden, The Netherlands). Tumor and metastasis samples were randomly processed. Genome-wide mRNA expression analyses of tumor samples, metastases and RCC cell lines were performed using the Human Transcriptome Array 2.0 (Affymetrix). In brief, RNA was purified from fresh-frozen ccRCC and metastasis tissue, as well as cell lines using the mirVana™ miRNA Isolation Kit (Life Technologies) and microarrays were processed according to manufacturer’s procedure (Affymetrix). The accession number for data of metastases and primary ccRCC, as well as cell lines, at the European Genome-phenome Archive (EGA) (www.ebi.ac.uk/ega/home), which is hosted by the EBI and the CRG, is EGAS00001001176. Further details about data analyses are provided in Supplementary Methods.

### DNA methylation, mRNA, and protein expression of OCT2/SLC22A2

For quantitative DNA methylation analyses of *SLC22A2*, matrix-assisted laser desorption ionization time-of-flight mass spectrometry (MALDI-TOF MS) was applied as described^26^. mRNA levels were quantified using TaqMan technology and OCT2 protein was quantified through Western Blot technology using a validated polyclonal antibody against OCT2 as described previously^26^. Tissue microarray sections were processed and immunostained as previously described^25^,^54^. Details see Supplementary Methods.

### Statistical analyses

Statistical analyses were performed with R-3.1.1 (http://www.r-project.org)^55^ or GraphPad Prism 5.0 (GraphPad Software, Inc., USA). *P*-values were adjusted for multiple testing by the Benjamini-Hochberg procedure^56^. All statistical tests were two-sided. Statistical significance level was defined as 5%. See Supplementary Data for details.

## Additional Information

**How to cite this article**: Winter, S. *et al*. Methylomes of renal cell lines and tumors or metastases differ significantly with impact on pharmacogenes. *Sci. Rep.* **6**, 29930; doi: 10.1038/srep29930 (2016).

## Supplementary Material

Supplementary Information

## Figures and Tables

**Table 1:** Patient cohort.

**Characteristics of patients/primary tumors**	**levels/summary statistics**	**Patients (n = 17) with ccRCC metastases**		**Patients (n = 34) with primary ccRCCs**	
		**no.**	**%**	**no.**	**%**
Sex	male	14	82.4	26	76.5
	female	3	17.6	8	23.5
Age (years) at diagnosis of primary RCC	median (range)	63 (33–75)		63 (35–90)	
T	1a	0	0	1	2.9
	1b	6	35.3	10	29.4
	2	0	0	2	5.9
	3a	6	35.3	12	35.3
	3b	4	23.5	9	26.5
	4	1	5.9	0	0
N^#^	0	11	64.7	27	79.4
	1	2	11.8	4	11.8
	2	4	23.5	3	8.8
M^#^	0	13	76.7	23	67.6
	1	4	23.5	11	32.4
G	1	1	5.9	3	8.8
	2	9	52.9	20	58.8
	3/4	7	41.2	11	32.4
Primary tumor size (cm)	median (range)	6.5 (1.5–14)		6.0 (1.5–16)	
Follow-up time (years) from date of diagnosis of primary ccRCC	median (range)	3.0 (0.14–17.7)		2.1 (0.09–8.6)	
Cancer-related death	no	12	70.6	17	50.0
	yes	5	29.4	17	50.0
Overall survival	alive	12	70.6	15	44.1
	dead	5	29.4	19	55.9
**Characteristics of metastasis/local recurrence specimens (n = 20)**	**levels/summary statistics**	**no.**		**%**	
Metastatic site	lymph node	7		35.0	
	adrenal gland	3		15.0	
	abdominal wall/cutaneous	2		10.0	
	local recurrence	2		10.0	
	ileum	1		5.0	
	liver	1		5.0	
	lung	1		5.0	
	pancreas	1		5.0	
	soft tissue (shoulder)	1		5.0	
	soft tissue (thorax)	1		5.0	
Age (years) at metastasis resection	median (range)	65 (34–77)			
Metastasis	metachronous	13		65.0	
	synchronous	7		35.0	
Years from diagnosis of primary RCC to metastasis resection	median (range)	1.2 (−0.08–17.7)*			
Follow-up time (years) from date of metastasis resection	median (range)	1.2 (0–7.6)			
Systemic therapy before metastasis resection	no	16		80.0	
	yes	4		20.0	

Characteristics of patients with primary ccRCC (n = 34) and of metastatic patients (n = 17) from which a total of 20 metastases^§^ were analysed in the present study. Abbreviations: T, primary tumor; N, regional lymph nodes; M, distant metastasis; G, grading.

^#^N, M refers to status at primary surgery. ^§^In three cases two metastases from the same patient were analyzed.

^*^In one case resection of the metastasis was performed 31 days before diagnosis and surgery of the primary ccRCC.

**Table 2:** Correlation of DNA methylation and gene expression.

**Gene**	**Chr.**	**Significantly negatively correlated CpG sites within gene**^**#**^	**No. of CpG sites**	Spearman’s correlation coefficient*
ADME Genes				
ABCC3	chr17	**cg01054938**, cg25928474	2	−0.5
ABCG1	chr21	cg02241241, cg21410080, cg27243685, cg02370100, **cg07397296**, cg00222799	6	−0.58
ALDH8A1	chr6	**cg02396253**, **cg20352402**	2	−0.71
CHST3	chr10	cg06370069, **cg09686390**	2	−0.64
CYP7B1	chr8	cg24990212, cg15160198, cg03535659, cg09975850, cg19424531, cg00054210, **cg01510388**	7	−0.68
FMO1	chr1	**cg13081014**	1	−0.64
GPX3	chr5	cg22005145, **cg18849169**, cg08891071, cg17820459, cg12684668	5	−0.6
GPX7	chr1	cg02453146, cg09161043, **cg11953272**, cg12640469, cg16557944, **cg18087326**, cg20950465, cg22129364, cg23272399, cg26251270	10	−0.73
GSTT1	chr22	**cg01238044**, cg11478607	2	−0.76
HNMT	chr2	cg02906939, **cg15441973**, cg07757007	3	−0.72
SLC22A2	chr6	cg07601258, cg02490934, cg13717233, cg19561774, cg19627213, cg04294894, **cg21755969**, cg02666489, cg25545503, cg07026448	10	−0.77
SLC28A1	chr15	cg12302621, **cg08271842**, cg10680235, cg22776451, cg22294181	5	−0.79
SLC2A5	chr1	cg03679305, **cg12763828**, **cg00310940**, cg07787240	4	−0.61
SLC6A6	chr3	**cg07438246**	1	−0.54
SLC7A8	chr14	**cg15527515**	1	−0.66
SLCO2A1	chr3	cg02496728, cg07780818, **cg05430989**	3	−0.63
SOD2	chr6	**cg06346099**	1	−0.61
SULF1	chr8	**cg07073960**	1	−0.39
Drug Target Genes				
CCND2	chr12	cg12382902, cg18584387, cg21057429, cg25454116, **cg26864834**	5	−0.61
CDH1	chr16	cg01857829, cg09406989, cg11667754, cg17655614, **cg20716119**, cg24765079	6	−0.67
CDK6	chr7	cg11998200, **cg19103429**, cg25156198	3	−0.56
DDR2	chr1	cg17217691, cg21539842, cg21880888, cg22740835, **cg23028772**	5	−0.58
ERBB3	chr12	cg00907267, **cg04794420**, cg10869879, cg11835619	4	−0.74
ERG	chr21	**cg06032349**	1	−0.67
KDR	chr4	**cg01340163**, cg07893544, cg10740902, cg18177533, cg21161891	5	−0.54
PDGFRB	chr5	**cg04173992**, cg11042320, cg14051336, cg25613180	4	−0.64

Pharmacogenes^§^ showing significant negative correlations between gene expression and DNA methylation in primary ccRCC (n = 34) and cell lines (n = 5). Abbreviations: Chr., chromosome; No., number

^§^Only pharmacogenes differing significantly and relevantly between primary ccRCC and cell lines in gene expression and DNA methylation were considered (details see text).

^#^CpG sites showing the minimal correlation coefficient are printed in bold.

^*^In case of several significantly negative correlated CpG sites, the minimal correlation coefficient is given.
