# Supplementary material for: Methylomes of renal cell lines and tumors or metastases differ significantly with impact on pharmacogenes
Source: Sci Rep. 2016 Jul 20;6:29930. doi: 10.1038/srep29930 (PMC4951699; doi:10.1038/srep29930)
Supplement: Supplementary Information [file srep29930-s1.pdf]

Methylomes of renal cell lines and tumors or metastases differ significantly with impact on pharmacogenes

Supplementary Data

Stefan Winter[1], Pascale Fisel[1], Florian Büttner[1,2], Steffen Rausch[3], Debora D`Amico[1], Jörg Hennenlotter[3], Stephan Kruck[3], Anne T. Nies[1], Arnulf Stenzl[3], Kerstin Junker[4], Marcus Scharpf[5], Ute Hofmann[1], Heiko van der Kuip[1], Falko Fend[5], German Ott[6], Abbas Agaimy[7], Arndt Hartmann[7], Jens Bedke[2,3], Matthias Schwab[1,2,8], and Elke Schaeffeler[1]

[1]Dr. Margarete Fischer-Bosch Institute of Clinical Pharmacology, Stuttgart, Germany and University of Tuebingen, Auerbachstr.112, 70376 Stuttgart, Germany; [2]German Cancer Consortium (DKTK) and German Cancer Research Center (DKFZ), Heidelberg, Germany; [3]Department of Urology, University Hospital Tuebingen, Hoppe-Seyler-Str. 3, 72076 Tuebingen, Germany; [4]Department of Urology and Pediatric Urology, Saarland University Medical Center and Saarland University Faculty of Medicine, Kirrberger Straße, 66424 Homburg/Saar, Germany; [5]Institute of Pathology and Neuropathology, University Hospital Tuebingen, Liebermeisterstr. 8, 72076 Tuebingen, Germany; [6]Department of Clinical Pathology, Robert-Bosch-Krankenhaus, Auerbachstr. 110, 70376 Stuttgart, Germany; [7]Institute of Pathology, University Erlangen-Nürnberg, Krankenhausstr. 8-10, 91054 Erlangen, Germany; [8]Department of Clinical Pharmacology, University Hospital Tuebingen, Auf der Morgenstelle 8, 72076 Tuebingen, Germany.

Supplementary Figures S1-9

Supplementary Tables S1-5

Supplementary Methods and References

Supplementary Figure S1

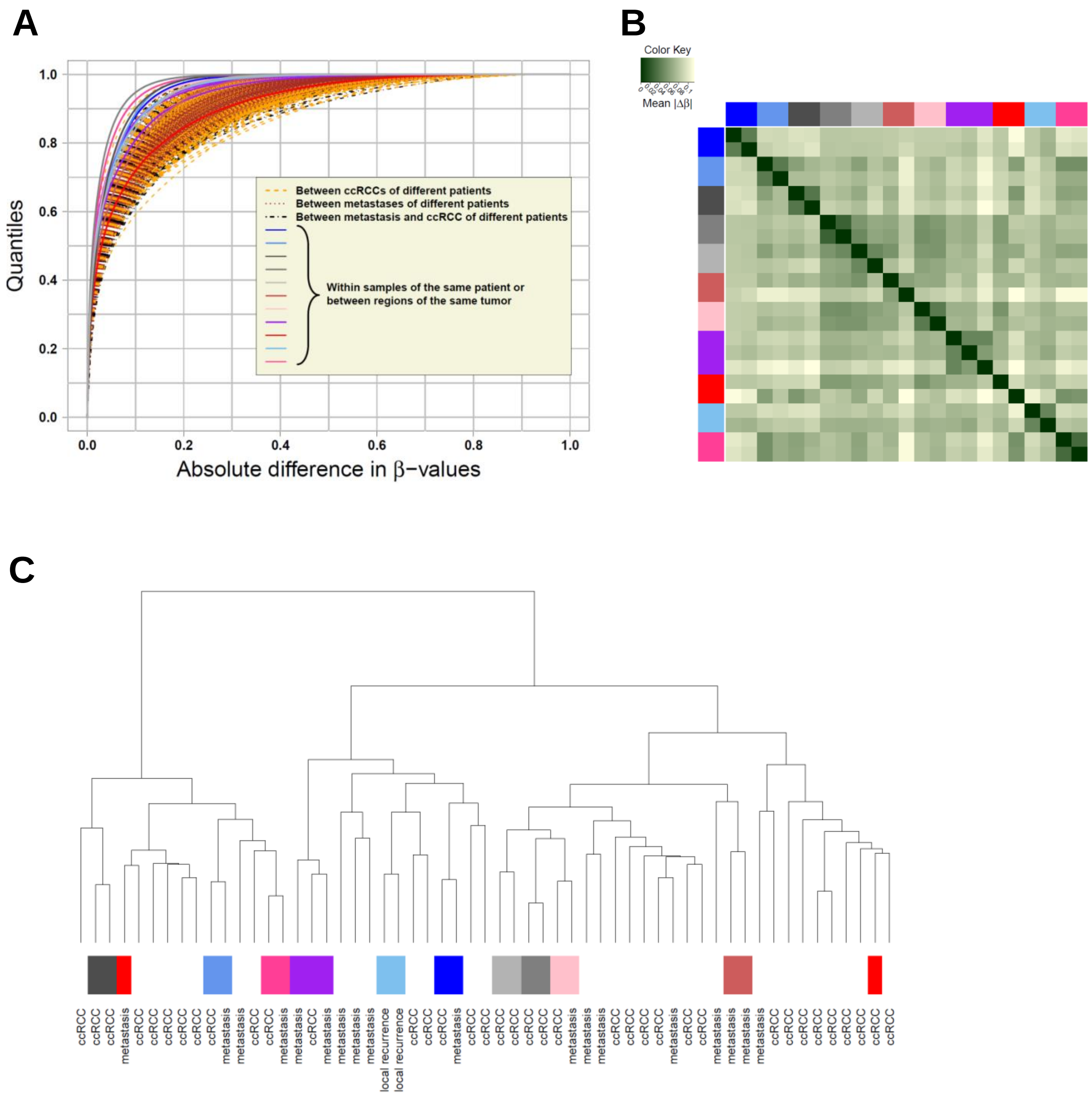

DNA methylation in metastases and primary ccRCC. A) Empirical distribution functions of absolute differences in DNA methylation levels (β-values) for all pairwise comparisons of the samples in our cohort. Curves for samples from the same patient are displayed as solid lines and in the same colors as in B and C. **B)** Heatmap showing mean absolute differences in DNA methylation levels (β-values). Samples from the same patient are marked in the same colors as in B and C in the vertical and horizontal side bar. Mean absolute differences in DNA methylation levels (Mean |Δβ|) are color-coded as indicated. **C)** Hierarchical cluster analysis of ccRCC (n=34), distant metastases (n=18) and local recurrences (n=2). DNA methylation profile in local recurrences of ccRCC reflects DNA methylation in primary ccRCC and distant metastases (samples from the same patient are marked by identical colors). Of note, for three tumors DNA methylation was investigated in two tumor regions (marked in identical grey shade).

Supplementary Figure S2

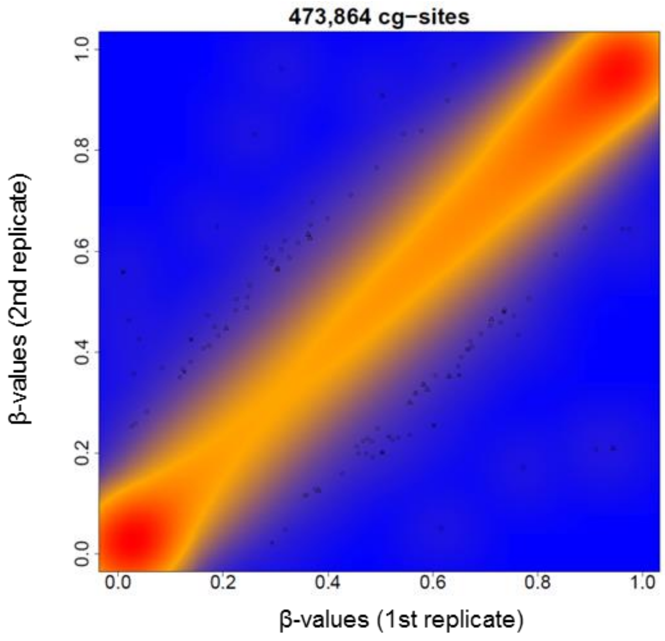

Genome-wide DNA methylation analyses using Illumina Human Methylation 450 BeadChip in technical replicates. DNA methylation levels of two replicates were highly correlated (r_S=0.99) and showed low absolute differences (mean absolute difference in β-values=0.020).

Supplementary Figure S3

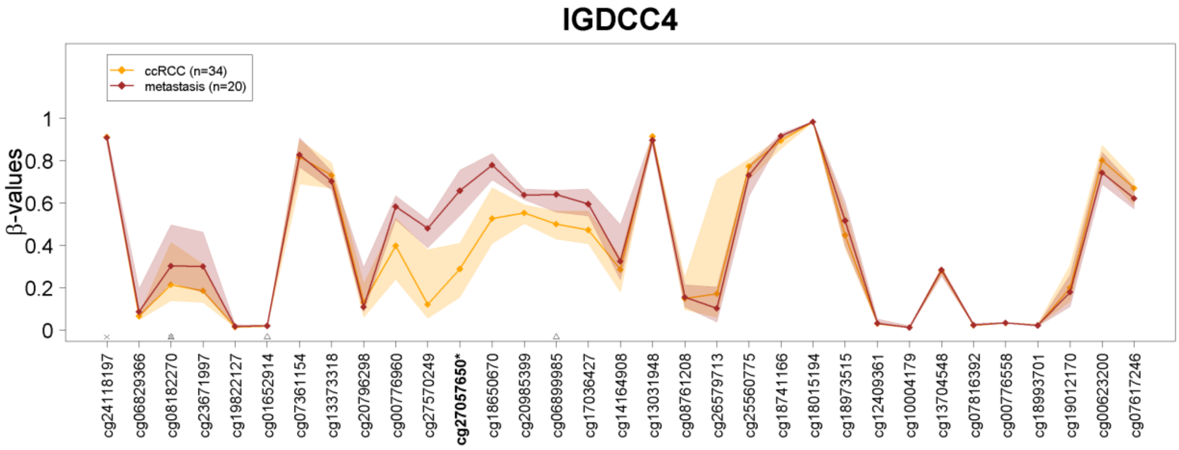

Differentially methylated CpG-sites in the *IGDCC4* gene. DNA methylation in the *IGDCC4* gene in metastasis (marked in brown) and in primary ccRCC (marked in orange) quantified using Infinium HumanMethylation450 BeadChip. Diamonds represent median methylation levels at single CpG sites; shaded areas are defined by 25% and 75% quantiles (cross-reactive probes are indicated by x; probes annotated to have non-rare SNPs within 50bp of probed CpG by Δ). Identified CpG site (see Supplementary Table S2) is marked in bold.

Supplementary Figure S4

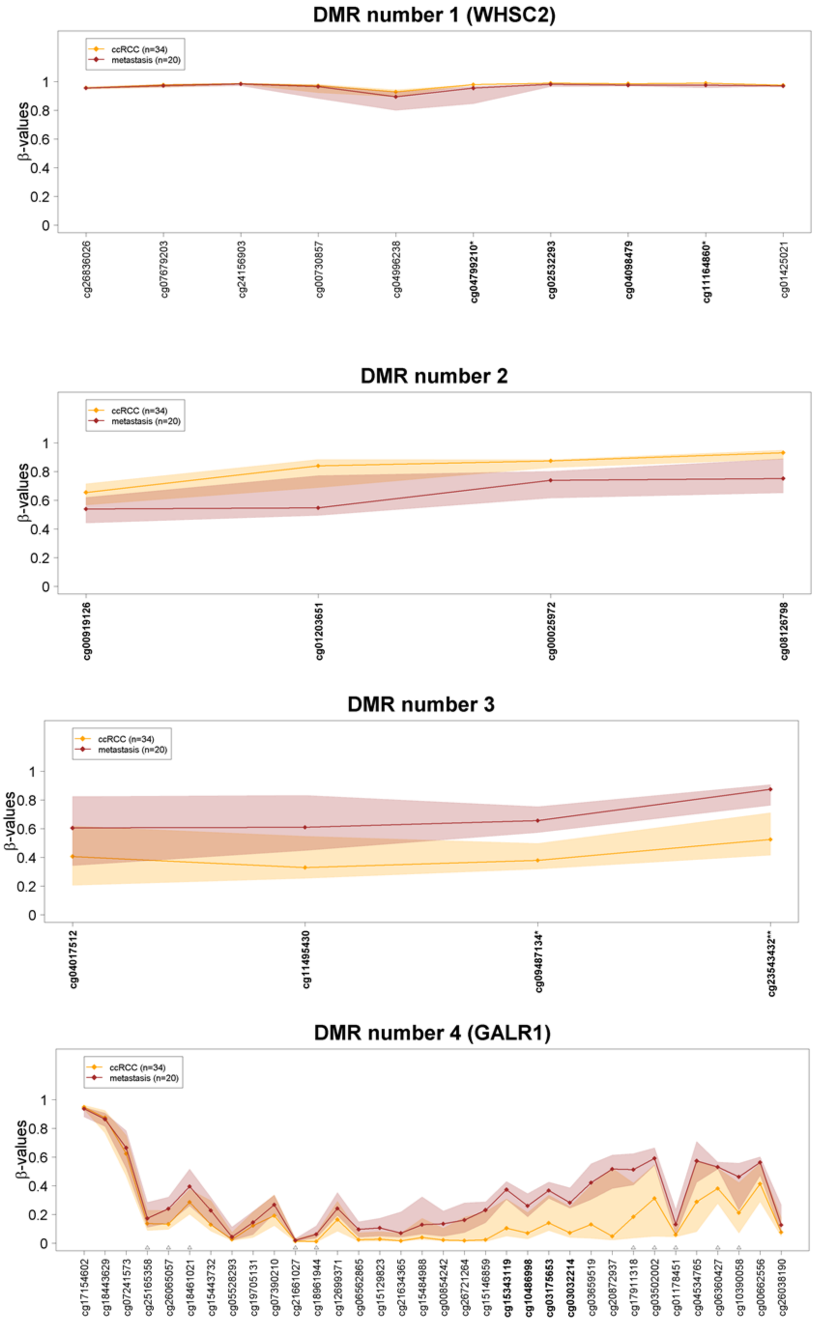

Differentially methylated regions (DMR) (results from Supplementary Table S3). DNA methylation in metastasis (marked in brown) and in primary ccRCC (marked in orange) quantified using Infinium HumanMethylation450 BeadChip. Diamonds represent median methylation levels at single CpG sites; shaded areas are defined by 25% and 75% quantiles (cross-reactive probes are indicated by x; probes annotated to have non-rare SNPs within 50bp of probed CpG by Δ). Identified DMRs (see Supplementary Table S3) are marked in bold.

Supplementary Figure S5

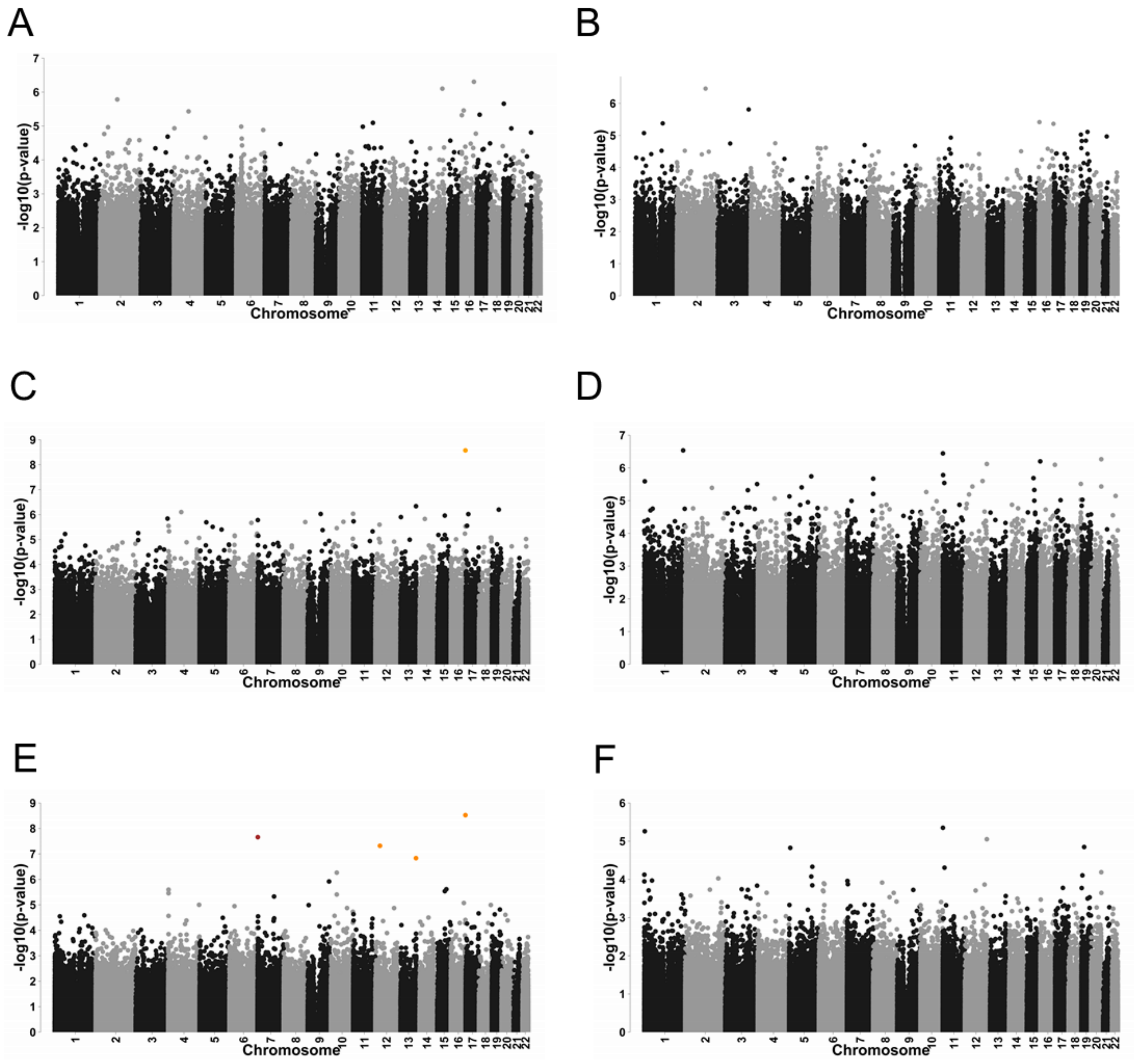

Manhattan plots showing results of linear mixed model analyses of differentially methylated cg-probes, comparing **A)** lymph node metastases (n=7) and metastases in other organs (n=13); **B)** metachronous (n=13) and synchronous (n=7) metastases; **C)** metastases (n=20) and non-metastatic ccRCC tumors at time of surgery (M0, N0; n=20); **D)** metastases (n=20) and primary RCCs, for which no metastasis occurred during follow-up (n=13); **E)** metastases (n=20) and primary RCCs, for which one or more metastases occurred during follow-up (n=21); **F)** primary RCCs for which no metastasis occurred (n=13) and for which one or more metastases occurred during follow-up (n=21). Analyses are based on 473,864 cg-probes (including cross-reactive probes and probes that are impaired by the presence of SNPs). Cg-probes with Benjamini-Hochberg adjusted *P*-value ≤ 5%, that are hypomethylated are marked in orange, hypermethylated cg-probes are marked in brown.

Supplementary Figure S6A

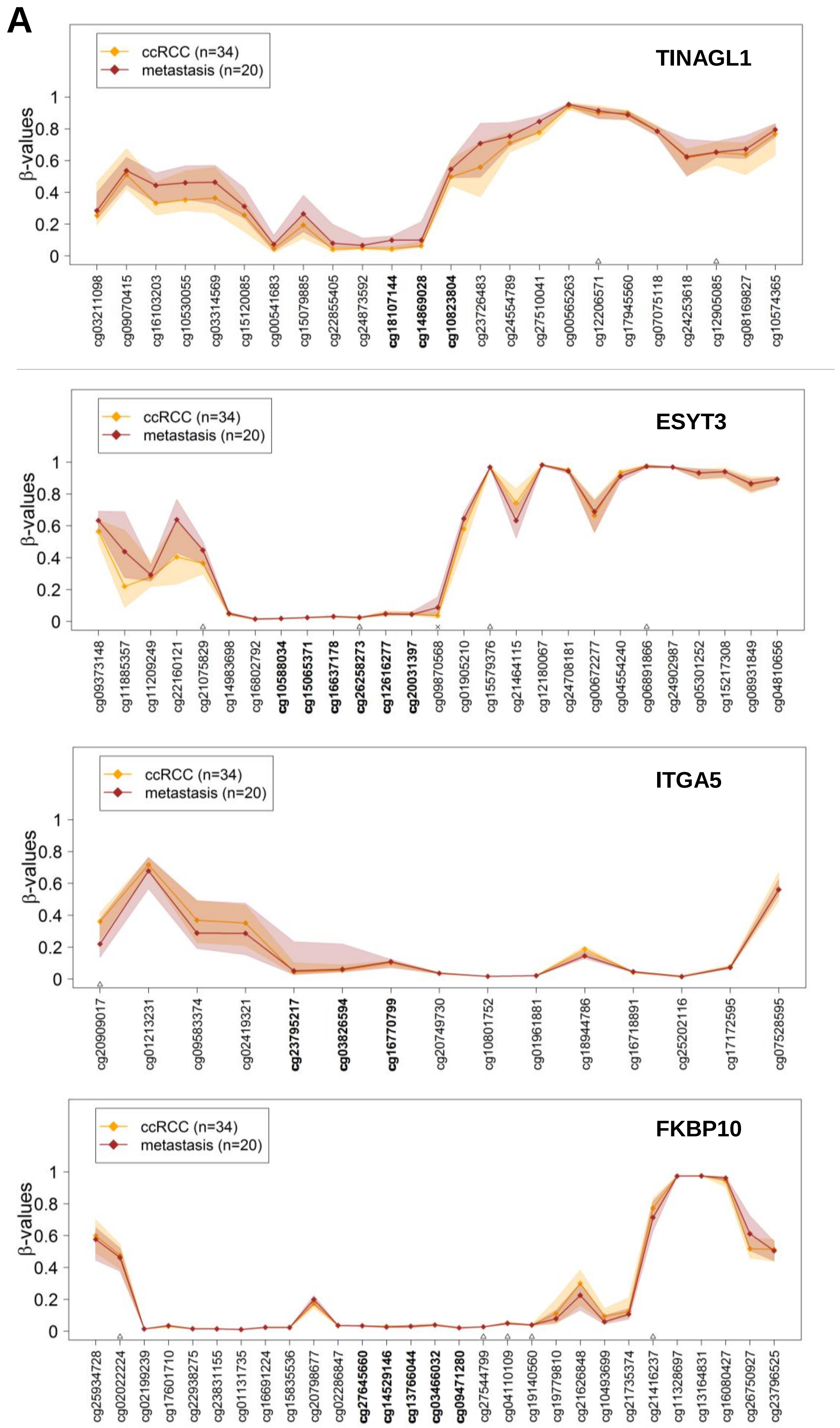

Supplementary Figure S6B-C

B

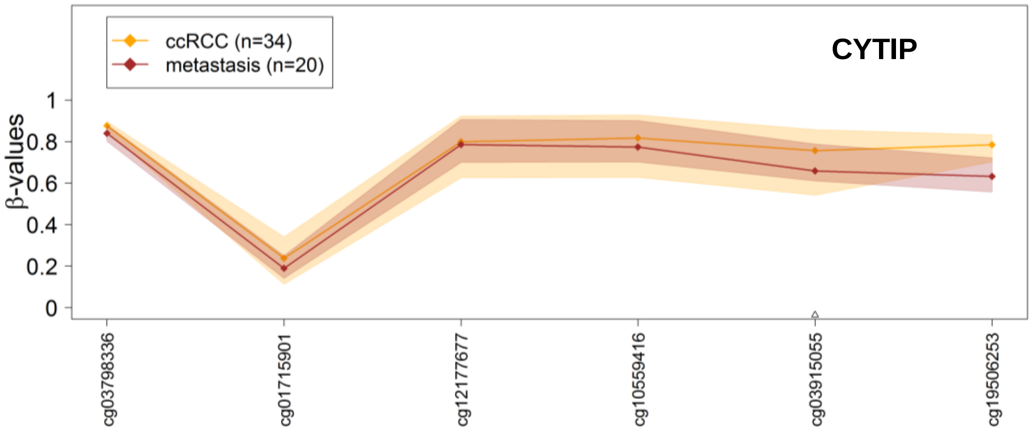

C

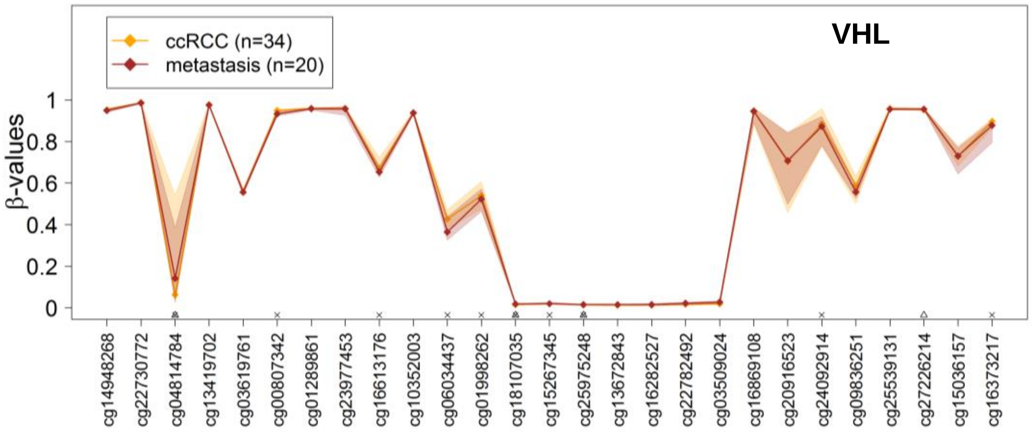

DNA methylation profiles of selected candidate genes. A-C) DNA methylation in metastasis (marked in brown) and in primary ccRCC (marked in orange) was quantified using Infinium HumanMethylation450 Bead Chip. Diamonds represent median methylation levels at single CpG sites; shaded areas are defined by 25% and 75% quantiles (cross-reactive probes are indicated by x; probes annotated to have non-rare SNPs within 50bp of probed CpG by Δ). No significant differences in DNA methylation were observed between primary ccRCC and metastases for *TINAGL1*, *ESYT3, ITGA5, FKBP10, CYTIP* or *VHL* after adjustment for multiple testing.

Supplementary Figure S7

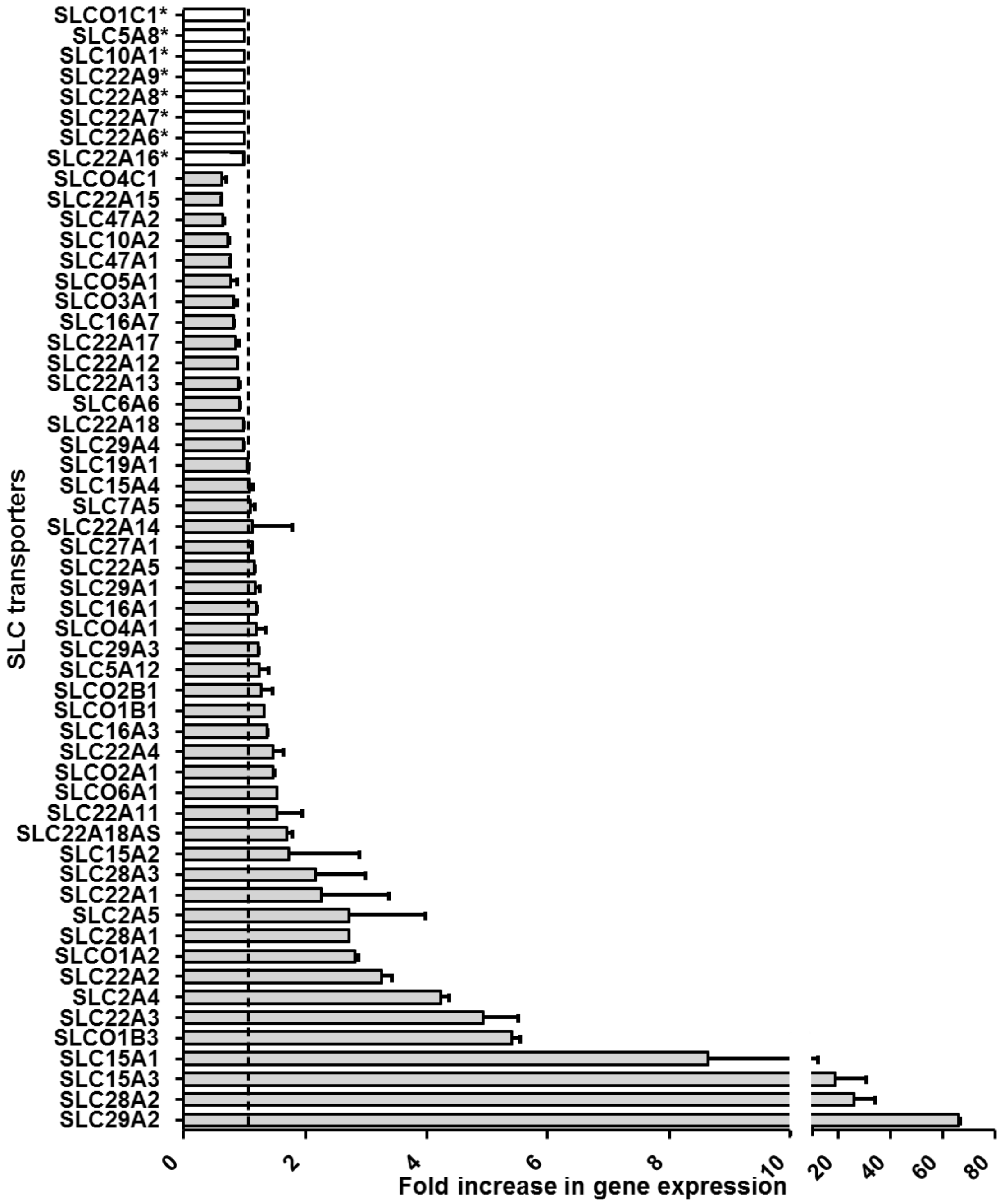

Effect of decitabine treatment on expression of 55 SLC transporters in Caki-2 cells. Fold increase in expression was calculated compared to untreated control cells. *Expression levels for these SLC transporters could be determined neither in treated cells nor in untreated cells.

Supplementary Figure S8

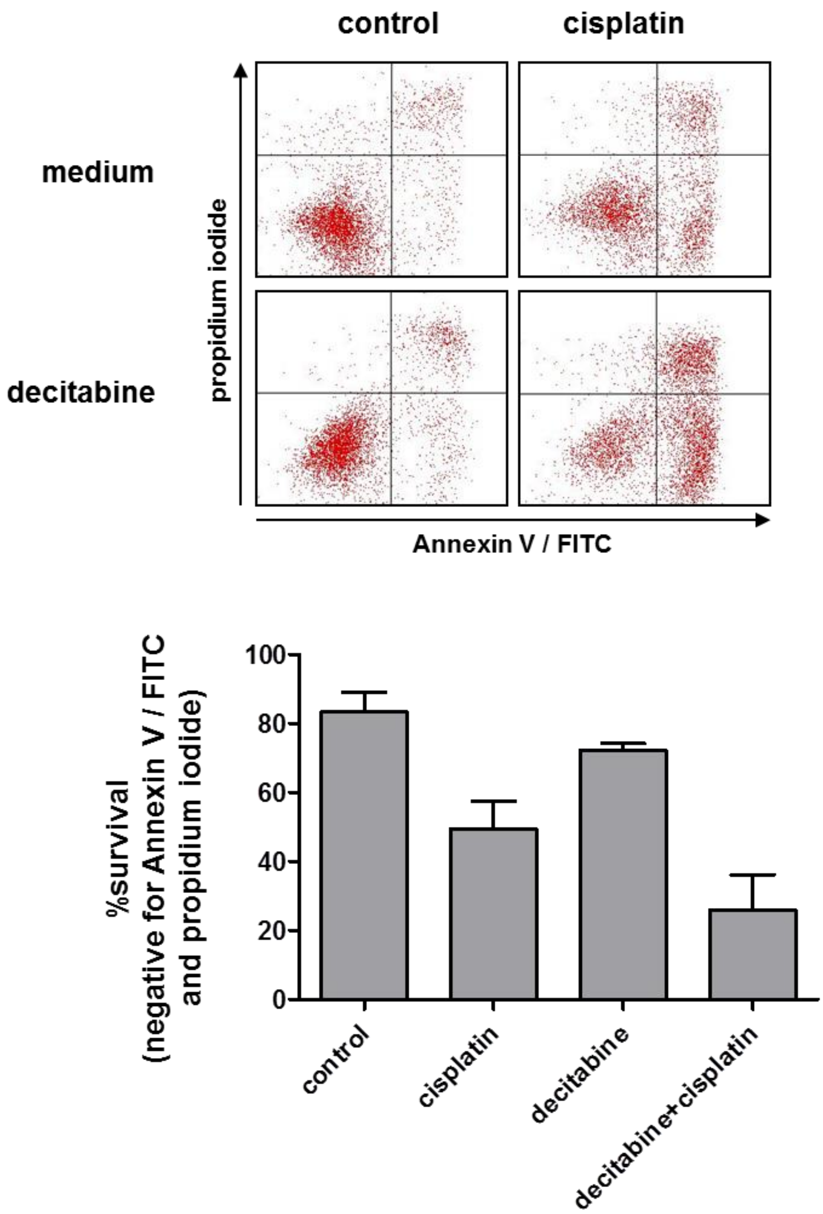

Effect of decitabine and cisplatin on apoptosis in Caki-2 cells.
Cells were stained with Annexin V / FITC and propidium iodide and analyzed by flow cytometry. Representative results of one experiment are displayed in the upper panel. Bar graph shows results of three independent experiments (mean with SD). Treatment of Caki-2 cells with a combination of decitabine and cisplatin resulted in a significantly increased induction of apoptosis compared to non-treated control cells (P<0.001) or cells treated with cisplatin (P<0.01) or decitabine (P<0.001) alone. P-values were calculated using repeated measures ANOVA with Newman-Keuls Post Test for multiple comparison.

Supplementary Figure S9

A

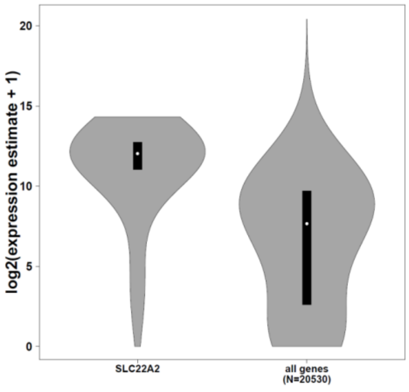

B

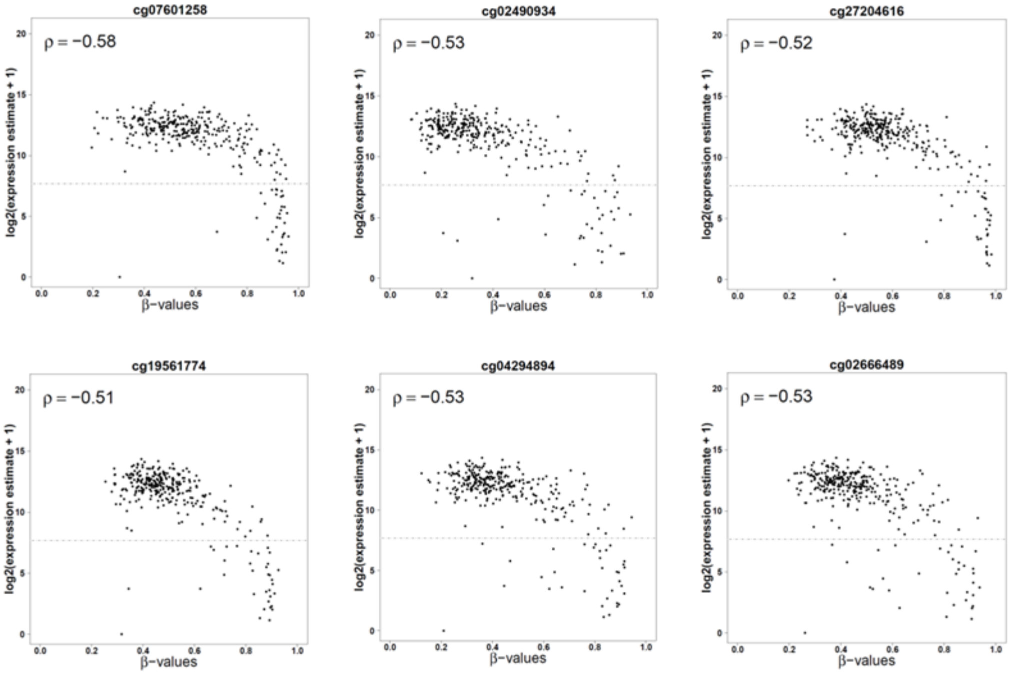

Expression and DNA methylation of SLC22A2/OCT2 in ccRCC tumors of the TCGA cohort. A) SLC22A2/OCT2 expression in 533 ccRCCs of the TCGA cohort. Only 12% display expression levels below median expression (=7.66, log2 (expression estimates+1)) of all genes (N= 20,350) analysed in the TCGA cohort. **B)** Exemplary data of six CpG sites, which exhibited highly significant inverse correlation (Spearman`s correlation coefficient<-0.5) between DNA methylation level and gene expression in ccRCC tissue of the TCGA cohort (n=318). These sites were located between the promoter region and first exon and intron region of *SLC22A2/OCT2*. Expression estimates denote RSEM expression estimates, normalized to set the upper quartile count at 1000 (details see Supplementary Methods).

Supplementary Table S1

Histopathological re-evaluation of samples with chromophobe RCC-like and papillary RCC-like DNA methylation pattern (identified by cluster analyses) of the TCGA cohort (diagnostic slides were used from http://cancer.digitalslidearchive.net/)

Sample (primary tumor)	Cluster	Histopathological re-evaluation by two independent renal tumor pathologists
TCGA-AK-3433	KICH-like	**non-ccRCC**; chromophobe RCC (DD: hybrid oncocytoma-chromophobe RCC)
TCGA-AK-3440	KICH-like	**non-ccRCC**; chromophobe RCC with microcystic and adenomatous arrangement and pigmentation (Hes et al. 2005)
TCGA-AK-3453	KICH-like	**non-ccRCC**; chromophobe RCC with microcystic and adenomatous arrangement and pigmentation (Hes et al. 2005) (DD: oncocytoma)
TCGA-B0-4696	KICH-like	**non-ccRCC**; chromophobe RCC
TCGA-B0-4699	KICH-like	**non-ccRCC**; chromophobe RCC
TCGA-B0-5083	KICH-like	**non-ccRCC**; chromophobe RCC
TCGA-B0-5117	KICH-like	**non-ccRCC**; chromophobe RCC
TCGA-B2-5636	KICH-like	**no conventional ccRCC**; RCC with features of angiomyoadenomatous tumor-like pattern (DD: ccRCC with smooth muscle stroma)
TCGA-B4-5378	KICH-like	**no conventional ccRCC**; RCC with features of angiomyoadenomatous tumor-like pattern (DD: mixed RCC with ccRCC and chromophobe RCC)
TCGA-B8-A54K	KICH-like	**no conventional ccRCC**; RCC with features of angiomyoadenomatous tumor-like pattern
TCGA-BP-4177*	KICH-like	**non-ccRCC**; tubulocystic RCC
TCGA-BP-4760	KICH-like	**no conventional ccRCC**; RCC with features of angiomyoadenomatous tumor-like pattern (DD: ccRCC with smooth muscle stroma)
TCGA-DV-5567	KICH-like	**no conventional ccRCC**; RCC with features of angiomyoadenomatous tumor-like pattern (DD: ccRCC with smooth muscle stroma)
TCGA-DV-5576	KICH-like	**no conventional ccRCC**; papillary RCC with clear cells
TCGA-DV-A4VZ	KICH-like	**no conventional ccRCC**; ccRCC with smooth muscle stroma
TCGA-B0-4688	KIRP-like	**non-ccRCC**; chromophobe RCC (DD: hybrid oncocytoma-chromophobe RCC)
TCGA-B0-5100	KIRP-like	**no conventional ccRCC**; RCC with features of angiomyoadenomatous tumor-like pattern
TCGA-A3-3385	KIRP-like	**ccRCC**
TCGA-B0-5098	KIRP-like	**no conventional ccRCC**; RCC (high grade), partially sarcomatoid, fumarate hydratase-deficient-RCC (DD: papillary RCC, Type II)
TCGA-B0-5707	KIRP-like	**non-ccRCC**; renal adenoleiomyomatous tumor (DD: tubulocystic RCC)
TCGA-B8-4621	KIRP-like	**no conventional ccRCC**; papillary RCC with clear cells or clear cell papillary RCC
TCGA-B8-5546	KIRP-like	**non-ccRCC**; renal adenoleiomyomatous tumor
TCGA-CJ-5681**	KIRP-like	**non-ccRCC**; renal adenoleiomyomatous tumor
TCGA-CZ-5469	KIRP-like	**ccRCC**
TCGA-EU-5907	KIRP-like	**non-ccRCC**; mainly adenomatoid, suspicion of chromophobe RCC

DD, differential diagnosis

*no diagnostic slide from http://cancer.digitalslidearchive.net was available, histological slides for re-evaluation were used from http://tcga.lbl.gov/biosig/tcgapatientlist.do?tumorType=KIRC&aliais=KIRC.

**no diagnostic slide from http://cancer.digitalslidearchive.net was available, histological slides for re-evaluation were used from https://tcga-data.nci.nih.gov/tcgafiles/ftp_auth/distro_ftpusers/anonymous/tumor/kirc/bcr/nationwidechildrens.org/diagnostic_images/slide_images/

Reference: Hes O, Vanecek T, Perez-Montiel DM, Alvarado Cabrero I, Hora M, Suster S, Lamovec J, Curik R, Mandys V, Michal M. Chromophobe renal cell carcinoma with microcystic and adenomatous arrangement and pigmentation—a diagnostic pitfall. Morphological, immunohistochemical, ultrastructural and molecular genetic report of 20 cases. Virch Arch 2005;446:383-393

Supplementary Table S2 Cg-probes with Benjamin-Hochberg adjusted *P*-value ≤ 5% in linear mixed model analyses between metastases and primary ccRCCs, with correction for age, sex, and array batch plus consideration of multiple samples for eight patients

				**Median β-value[1] in**			**Analysis on 473,864 cg-sites[2]**		**Analysis on 348,721 cg-sites[2]**			
**Cg-probe**	**Chr**	**Assigned Gene (UCSC)**	**Relation to CpG Island**	**Metastases (n=20)**	**ccRCCs (n=34)**	**Difference in median β-values[1] (metastases - ccRCCs)**	**Unadjusted *P*-value**	**BH-adjusted *P*-value**	**Unadjusted *P*-value**	**BH-adjusted *P*-value**	**Cross-reactive probe[3]**	**Non-rare SNP within 50bp of probed CpG[4]**
cg08940169	16	ZFPM1	Island	0.912	0.973	*-0.061*	2.75E-10	0.00013	2.65E-10	0.0000925	NO	NO
cg02968407	7	-	Island	0.976	0.887	*0.089*	5.22E-09	0.00124	6.08E-09	0.00106	NO	NO
cg12444736	13	-	Island	0.786	0.917	**-0.131**	3.12E-08	0.00465	3.51E-08	0.00408	NO	NO
cg05637360	12	PPFIBP1	OpenSea	0.768	0.861	*-0.093*	3.92E-08	0.00465	-	-	YES	NO
cg23543432	10	-	OpenSea	0.874	0.525	**0.349**	8.84E-08	0.00837	8.96E-08	0.00781	NO	NO
cg27057650	15	IGDCC4	N_Shore	0.658	0.288	**0.37**	0.00000024	0.0184	0.000000242	0.0152	NO	NO
cg09487134	10	-	OpenSea	0.655	0.380	**0.275**	0.000000272	0.0184	0.000000261	0.0152	NO	NO
cg14360444	9	ZBTB34	OpenSea	0.977	0.984	-0.007	0.0000004	0.0237	0.000000429	0.0214	NO	NO
cg04799210	4	WHSC2	Island	0.954	0.979	-0.025	0.000000564	0.0289	0.000000576	0.0251	NO	NO
cg18581777	15	LMAN1L	OpenSea	0.912	0.952	-0.04	0.000000609	0.0289	0.000000726	0.0281	NO	NO
cg03120102	16	WWOX	OpenSea	0.963	0.973	-0.009	0.000000815	0.0351	-	-	YES	NO
cg11164860	4	WHSC2	Island	0.976	0.989	-0.013	0.00000108	0.0428	0.00000107	0.0374	NO	NO
cg19631815	12	DTX1	N_Shelf	0.975	0.982	-0.007	0.0000015	0.0541	0.00000169	0.0454	NO	NO
cg23634681	18	FBXO15	OpenSea	0.950	0.970	-0.02	0.0000016	0.0541	0.00000156	0.0454	NO	NO
cg00025972	4	-	OpenSea	0.738	0.875	**-0.137**	0.00000174	0.0548	0.00000162	0.0454	NO	NO

Abbreviations: Chr, Chromosome; BH, Benjamini-Hochberg; SNP, single nucleotide polymorphism; bp, base pairs

Absolute differences in median β-values ≥ 10% are marked in bold, between 5% and 10% in italic.

[1] Medians were calculated on batch-corrected β-values (vide Supplementary Methods)
[2] Analysis with or without filtering of cross-reactive probes and probes annotated to have non-rare SNPs within 50bp of probed CpG (see below and Supplementary Methods)
[3] Probe found by Chen et al. (2013) [15] to be cross-reactive with areas of the genome not at the site of interest
[4] SNP within 50bp of probed CpG in the European population (1000 Genomes) with MAF>1%

Supplementary Table S3 DMR between metastases of ccRCC and primary ccRCC using relaxed thresholds (details of statistical analyses see Supplementary Methods)

							**Analysis on 473,864 cg-sites[2]**		**Analysis on 348,721 cg-sites[2]**	
**DMR No.**	**Chr**	**DMR Start – End (chr. Pos.)**	**Assigned Gene (UCSC)**	**Relation to CpG Island**	**Cg-probes in DMR**	**Difference in median β-values[1] (metastases - ccRCC)**	**BH-adjusted *P*-value**	**ChAMP DMR *P*-value**	**BH-adjusted P-value**	**ChAMP DMR *P*-value**
**1**	4	1994682 - 1994950	*WHSC2*	Island	cg04799210	-0.025	*0.029*	0.003	*0.025*	0.004
					cg02532293	-0.008	*0.112*		*0.090*	
					cg04098479	-0.009	*0.149*		*0.137*	
					cg11164860	-0.013	*0.043*		*0.037*	
**2**	10	35194787 - 35196605	-	Open Sea	cg04017512	**0.198**	0.407	0.003	0.418	0.005
					cg11495430	**0.279**	0.155		*0.144*	
					cg09487134	**0.275**	*0.018*		*0.015*	
					cg23543432	**0.349**	*0.008*		*0.008*	
**3**	4	187761071 - 187763620	-	Open Sea	cg00919126	**-0.116**	0.350	-	0.357	0.034
					cg01203651	**-0.294**	0.286		0.279	
					cg00025972	**-0.137**	*0.055*		*0.045*	
					cg08126798	**-0.180**	0.167		*0.144*	
**4**	18	74961759 - 74961824	*GALR1*	Island	cg15343119	**0.269**	*0.147*	-	*0.130*	0.043
					cg10486998	**0.189**	0.262		0.256	
					cg03175653	**0.227**	0.155		*0.144*	
					cg03032214	**0.209**	0.205		0.196	

Abbreviations: DMR, Differentially methylated region; Chr, Chromosome; BH, Benjamini-Hochberg

Absolute differences in median β-values ≥ 10% are marked in bold, BH-adjusted *P*-value ≤ 15% in italic.

[1] Medians were calculated on batch-corrected β-values (see Supplementary Methods)
[2] Analysis without or after filtering of cross-reactive probes and probes annotated to have non-rare SNPs within 50bp of probed CpG (see Supplementary Methods)

Supplementary Table S4

List of ADME genes:

Gene Symbol	Full Gene Name	Class	Type
ABCB1	ATP-binding cassette, sub-family B (MDR/TAP), member 1	Transporter	Core
ABCC2	ATP-binding cassette, sub-family C (CFTR/MRP), member 2	Transporter	Core
ABCG2	ATP-binding cassette, sub-family G (WHITE), member 2	Transporter	Core
CYP1A1	cytochrome P450, family 1, subfamily A, polypeptide 1	Phase I	Core
CYP1A2	cytochrome P450, family 1, subfamily A, polypeptide 2	Phase I	Core
CYP2A6	cytochrome P450, family 2, subfamily A, polypeptide 6	Phase I	Core
CYP2B6	cytochrome P450, family 2, subfamily B, polypeptide 6	Phase I	Core
CYP2C19	cytochrome P450, family 2, subfamily C, polypeptide 19	Phase I	Core
CYP2C8	cytochrome P450, family 2, subfamily C, polypeptide 8	Phase I	Core
CYP2C9	cytochrome P450, family 2, subfamily C, polypeptide 9	Phase I	Core
CYP2D6	cytochrome P450, family 2, subfamily D, polypeptide 6	Phase I	Core
CYP2E1	cytochrome P450, family 2, subfamily E, polypeptide 1	Phase I	Core
CYP3A4	cytochrome P450, family 3, subfamily A, polypeptide 4	Phase I	Core
CYP3A5	cytochrome P450, family 3, subfamily A, polypeptide 5	Phase I	Core
DPYD	dihydropyrimidine dehydrogenase	Phase I	Core
GSTM1	glutathione S-transferase M1	Phase II	Core
GSTP1	glutathione S-transferase pi	Phase II	Core
GSTT1	glutathione S-transferase theta 1	Phase II	Core
NAT1	N-acetyltransferase 1 (arylamine N-acetyltransferase)	Phase II	Core
NAT2	N-acetyltransferase 2 (arylamine N-acetyltransferase)	Phase II	Core
SLC15A2	solute carrier family 15 (H+/peptide transporter), member 2	Transporter	Core
SLC22A1	solute carrier family 22 (organic cation transporter), member 1	Transporter	Core
SLC22A2	solute carrier family 22 (organic cation transporter), member 2	Transporter	Core
SLC22A6	solute carrier family 22 (organic anion transporter), member 6	Transporter	Core
SLCO1B1	solute carrier organic anion transporter family, member 1B1	Transporter	Core
SLCO1B3	solute carrier organic anion transporter family, member 1B3	Transporter	Core
SULT1A1	sulfotransferase family, cytosolic, 1A, phenol-preferring, member 1	Phase II	Core
TPMT	thiopurine S-methyltransferase,	Phase II	Core
UGT1A1	UDP glucuronosyltransferase 1 family, polypeptide A1	Phase II	Core
UGT2B15	UDP glucuronosyltransferase 2 family, polypeptide B15	Phase II	Core
UGT2B17	UDP glucuronosyltransferase 2 family, polypeptide B17	Phase II	Core
UGT2B7	UDP glucuronosyltransferase 2 family, polypeptide B7	Phase II	Core
ABCB8	ATP-binding cassette, sub-family B (MDR/TAP), member 8	Transporter	Extended
ABCC12	ATP-binding cassette, sub-family C (CFTR/MRP), member 12	Transporter	Extended
ABCC3	ATP-binding cassette, sub-family C (CFTR/MRP), member 3	Transporter	Extended
ABCC4	ATP-binding cassette, sub-family C (CFTR/MRP), member 4	Transporter	Extended
AHR	aryl hydrocarbon receptor	Modifier	Extended
ALDH4A1	aldehyde dehydrogenase 4 family, member A1	Phase I	Extended
ALDH5A1	aldehyde dehydrogenase 5 family, member A1	Phase I	Extended
ALDH6A1	aldehyde dehydrogenase 6 family, member A1	Phase I	Extended
CES1	carboxylesterase 1 (monocyte/macrophage serine esterase 1)	Phase I	Extended
CES2	carboxylesterase 2 (intestine, liver)	Phase I	Extended
CYP7A1	cytochrome P450, family 7, subfamily A, polypeptide 1	Phase I	Extended
EPHX1	epoxide hydrolase 1, microsomal (xenobiotic)	Phase I	Extended
FMO3	flavin containing monooxygenase 3	Phase I	Extended
GSTA1	glutathione S-transferase A1	Phase II	Extended
GSTA2	glutathione S-transferase A2	Phase II	Extended
GSTA3	glutathione S-transferase A3	Phase II	Extended
GSTA4	glutathione S-transferase A4	Phase II	Extended
GSTA5	glutathione S-transferase A5	Phase II	Extended
GSTM2	glutathione S-transferase M2 (muscle),glutathione S-transferase M4	Phase II	Extended
GSTM3	glutathione S-transferase M3 (brain)	Phase II	Extended
GSTM4	glutathione S-transferase M4	Phase II	Extended

GSTO1	glutathione S-transferase omega 1,glutathione S-transferase omega 2	Phase II	Extended
GSTO2	glutathione S-transferase omega 2	Phase II	Extended
GSTT2	glutathione S-transferase theta 2	Phase II	Extended
SLC10A1	solute carrier family 10 (sodium/bile acid cotransporter family), member 1	Transporter	Extended
SLC15A1	solute carrier family 15 (oligopeptide transporter), member 1	Transporter	Extended
SLC22A11	solute carrier family 22 (organic anion/cation transporter), member 11	Transporter	Extended
SLC22A8	solute carrier family 22 (organic anion transporter), member 8	Transporter	Extended
SLC7A5	solute carrier family 7 (cationic amino acid transporter, y+ system), member 5	Transporter	Extended
SLCO1A2	solute carrier organic anion transporter family, member 1A2	Transporter	Extended
SLCO2B1	solute carrier organic anion transporter family, member 2B1	Transporter	Extended
SULT1A2	sulfotransferase family, cytosolic, 1A, phenol-preferring, member 2	Phase II	Extended
SULT1A3	sulfotransferase family, cytosolic, 1A, phenol-preferring, member 3	Phase II	Extended
SULT1B1	sulfotransferase family, cytosolic, 1B, member 1	Phase II	Extended
UGT1A3	UDP glucuronosyltransferase 1 family, polypeptide A3	Phase II	Extended
UGT1A6	UDP glucuronosyltransferase 1 family, polypeptide A6	Phase II	Extended
UGT1A7	UDP glucuronosyltransferase 1 family, polypeptide A7	Phase II	Extended
UGT1A8	UDP glucuronosyltransferase 1 family, polypeptide A8	Phase II	Extended
UGT1A9	UDP glucuronosyltransferase 1 family, polypeptide A9	Phase II	Extended
UGT2A1	UDP glucuronosyltransferase 2 family, polypeptide A1	Phase II	Extended
UGT2B11	UDP glucuronosyltransferase 2 family, polypeptide B11	Phase II	Extended
UGT2B28	UDP glucuronosyltransferase 2 family, polypeptide B28	Phase II	Extended
UGT2B4	UDP glucuronosyltransferase 2 family, polypeptide B4	Phase II	Extended
ABCA1	ATP-binding cassette, sub-family A (ABC1), member 1	Transporter	Extended
ABCA4	ATP-binding cassette, sub-family A (ABC1), member 4	Transporter	Extended
ABCB11	ATP-binding cassette, sub-family B (MDR/TAP), member 11	Transporter	Extended
ABCB4	ATP-binding cassette, sub-family B (MDR/TAP), member 4	Transporter	Extended
ABCB5	ATP-binding cassette, sub-family B (MDR/TAP), member 5	Transporter	Extended
ABCB6	ATP-binding cassette, sub-family B (MDR/TAP), member 6	Transporter	Extended
ABCB7	ATP-binding cassette, sub-family B (MDR/TAP), member 7	Transporter	Extended
ABCC1	ATP-binding cassette, sub-family C (CFTR/MRP), member 1	Transporter	Extended
ABCC10	ATP-binding cassette, sub-family C (CFTR/MRP), member 10	Transporter	Extended
ABCC11	ATP-binding cassette, sub-family C (CFTR/MRP), member 11	Transporter	Extended
ABCC5	ATP-binding cassette, sub-family C (CFTR/MRP), member 5	Transporter	Extended
ABCC6	ATP-binding cassette, sub-family C (CFTR/MRP), member 6	Transporter	Extended
ABCC8	ATP-binding cassette, sub-family C (CFTR/MRP), member 8	Transporter	Extended
ABCC9	ATP-binding cassette, sub-family C (CFTR/MRP), member 9	Transporter	Extended
ABCG1	ATP-binding cassette, sub-family G (WHITE), member 1	Transporter	Extended
ADH1A	alcohol dehydrogenase 1A (class I), alpha polypeptide	Phase I	Extended
ADH1B	alcohol dehydrogenase IB (class I), beta polypeptide	Phase I	Extended
ADH1C	alcohol dehydrogenase 1C (class I), gamma polypeptide	Phase I	Extended
ADH4	alcohol dehydrogenase 4 (class II), pi polypeptide	Phase I	Extended
ADH5	alcohol dehydrogenase 5 (class III), chi polypeptide,methionyl aminopeptidase 1	Phase I	Extended
ADH6	alcohol dehydrogenase 6 (class V)	Phase I	Extended
ADH7	alcohol dehydrogenase 7 (class IV), mu or sigma polypeptide	Phase I	Extended
ALDH1A1	aldehyde dehydrogenase 1 family, member A1	Phase I	Extended
ALDH1A2	aldehyde dehydrogenase 1 family, member A2	Phase I	Extended
ALDH1A3	aldehyde dehydrogenase 1 family, member A3	Phase I	Extended
ALDH1B1	aldehyde dehydrogenase 1 family, member B1	Phase I	Extended
ALDH2	aldehyde dehydrogenase 2 family (mitochondrial)	Phase I	Extended
ALDH3A1	aldehyde dehydrogenase 3 family, memberA1	Phase I	Extended
ALDH3A2	aldehyde dehydrogenase 3 family, member A2	Phase I	Extended
ALDH3B1	aldehyde dehydrogenase 3 family, member B1	Phase I	Extended
ALDH3B2	aldehyde dehydrogenase 3 family, member B2	Phase I	Extended
ALDH7A1	aldehyde dehydrogenase 7 family, member A1	Phase I	Extended
ALDH8A1	aldehyde dehydrogenase 8 family, member A1	Phase I	Extended
ALDH9A1	aldehyde dehydrogenase 9 family, member A1	Phase I	Extended
AOX1	aldehyde oxidase 1	Phase I	Extended
ARNT	aryl hydrocarbon receptor nuclear translocator	Modifier	Extended
CBR1	carbonyl reductase 1	Phase I	Extended
CBR3	carbonyl reductase 3	Phase I	Extended
CDA	cytidine deaminase	Modifier	Extended
CYB5R3	cytochrome b5 reductase 3	Phase I	Extended

CYP11A1	cytochrome P450, family 11, subfamily A, polypeptide 1	Phase I	Extended
CYP11B1	cytochrome P450, family 11, subfamily B, polypeptide 1	Phase I	Extended
CYP11B2	cytochrome P450, family 11, subfamily B, polypeptide 2	Phase I	Extended
CYP17A1	cytochrome P450, family 17, subfamily A, polypeptide 1	Phase I	Extended
CYP1B1	cytochrome P450, family 1, subfamily B, polypeptide 1	Phase I	Extended
CYP20A1	cytochrome P450, family 20, subfamily A, polypeptide 1	Phase I	Extended
CYP21A2	cytochrome P450, family 21, subfamily A, polypeptide 2	Phase I	Extended
CYP24A1	cytochrome P450, family 24, subfamily A, polypeptide 1	Phase I	Extended
CYP26A1	cytochrome P450, family 26, subfamily A, polypeptide 1	Phase I	Extended
CYP27A1	cytochrome P450, family 27, subfamily A, polypeptide 1	Phase I	Extended
CYP2A13	cytochrome P450, family 2, subfamily A, polypeptide 13	Phase I	Extended
CYP2A7	cytochrome P450, family 2, subfamily A, polypeptide 7	Phase I	Extended
CYP2C18	cytochrome P450, family 2, subfamily C, polypeptide 18	Phase I	Extended
CYP2F1	cytochrome P450, family 2, subfamily F, polypeptide 1	Phase I	Extended
CYP2J2	cytochrome P450, family 2, subfamily J, polypeptide 2	Phase I	Extended
CYP39A1	cytochrome P450, family 39, subfamily A, polypeptide 1	Phase I	Extended
CYP3A43	cytochrome P450, family 3, subfamily A, polypeptide 43	Phase I	Extended
CYP3A7	cytochrome P450, family 3, subfamily A, polypeptide 7	Phase I	Extended
CYP4B1	cytochrome P450, family 4, subfamily B, polypeptide 1	Phase I	Extended
CYP4F11	cytochrome P450, family 4, subfamily F, polypeptide 11	Phase I	Extended
CYP51A1	cytochrome P450, family 51, subfamily A, polypeptide 1	Phase I	Extended
EPHX2	epoxide hydrolase 2, cytoplasmic	Phase I	Extended
FMO1	flavin containing monooxygenase 1	Phase I	Extended
FMO2	flavin containing monooxygenase 2	Phase I	Extended
FMO4	flavin containing monooxygenase 4	Phase I	Extended
FMO5	flavin containing monooxygenase 5	Phase I	Extended
GPX2	glutathione peroxidase 2 (gastrointestinal)	Phase I	Extended
GPX3	glutathione peroxidase 3 (plasma)	Phase I	Extended
GPX7	glutathione peroxidase 7	Phase I	Extended
GSR	glutathione reductase	Phase I	Extended
GSTK1	glutathione S-transferase kappa 1	Phase II	Extended
GSTM5	glutathione S-transferase M5	Phase II	Extended
GSTZ1	glutathione transferase zeta 1 (maleylacetoacetate isomerase)	Phase II	Extended
NNMT	nicotinamide N-methyltransferase	Phase II	Extended
NR1I2	nuclear receptor subfamily 1, group I, member 2	Modifier	Extended
NR1I3	nuclear receptor subfamily 1, group I, member 3	Modifier	Extended
PNMT	phenylethanolamine N-methyltransferase	Phase II	Extended
PON1	paraoxonase 1	Phase I	Extended
PON2	paraoxonase 2	Phase I	Extended
PON3	paraoxonase 3	Phase I	Extended
POR	P450 (cytochrome) oxidoreductase	Modifier	Extended
PPARD	peroxisome proliferative activated receptor, delta	Modifier	Extended
PPARG	peroxisome proliferative activated receptor, gamma	Modifier	Extended
RXRA	retinoid X receptor, alpha	Modifier	Extended
SLC10A2	solute carrier family 10 (sodium/bile acid cotransporter family), member 2	Transporter	Extended
SLC13A1	solute carrier family 13 (sodium/sulfate symporters), member 1	Transporter	Extended
SLC13A2	solute carrier family 13 (sodium-dependent dicarboxylate transporter), member 2	Transporter	Extended
SLC13A3	solute carrier family 13 (sodium-dependent dicarboxylate transporter), member 3	Transporter	Extended
SLC16A1	solute carrier family 16 (monocarboxylic acid Transporter), member 1	Transporter	Extended
SLC19A1	solute carrier family 19 (folate transporter), member 1	Transporter	Extended
SLC22A10	solute carrier family 22 (organic anion/cation transporter), member 10	Transporter	Extended
SLC22A12	solute carrier family 22 (organic anion/cation transporter), member 12	Transporter	Extended
SLC22A13	solute carrier family 22 (organic cation transporter), member 13	Transporter	Extended
SLC22A14	solute carrier family 22 (organic cation transporter), member 14	Transporter	Extended
SLC22A15	solute carrier family 22 (organic cation transporter), member 15	Transporter	Extended
SLC22A16	solute carrier family 22 (organic cation transporter), member 16	Transporter	Extended
SLC22A17	solute carrier family 22 (organic cation transporter), member 17	Transporter	Extended
SLC22A18	solute carrier family 22 (organic cation transporter), member 18	Transporter	Extended
SLC22A18AS	solute carrier family 22 (organic cation transporter), member 18 antisense	Transporter	Extended
SLC22A3	solute carrier family 22 (extraneuronal monoamine transporter), member 3	Transporter	Extended
SLC22A4	solute carrier family 22 (organic cation transporter), member 4	Transporter	Extended
SLC22A5	solute carrier family 22 (organic cation transporter), member 5	Transporter	Extended

SLC22A7	solute carrier family 22 (organic anion transporter), member 7	Transporter	Extended
SLC22A9	solute carrier family 22 (organic anion/cation transporter), member 9	Transporter	Extended
SLC27A1	solute carrier family 27 (fatty acid transporter), member 1	Transporter	Extended
SLC28A1	solute carrier family 28 (sodium-coupled nucleoside transporter), member 1	Transporter	Extended
SLC28A2	solute carrier family 28 (sodium-coupled nucleoside transporter), member 2	Transporter	Extended
SLC28A3	solute carrier family 28 (sodium-coupled nucleoside transporter), member 3	Transporter	Extended
SLC29A1	solute carrier family 29 (nucleoside Transporter), member 1	Transporter	Extended
SLC29A2	solute carrier family 29 (nucleoside Transporter), member 2	Transporter	Extended
SLC2A4	solute carrier family 2 (facilitated glucose transporter), member 4	Transporter	Extended
SLC2A5	solute carrier family 2 (facilitated glucose/fructose transporter), member 5	Transporter	Extended
SLC5A6	solute carrier family 5 (sodium-dependent vitamin transporter)	Transporter	Extended
SLC6A6	solute carrier family 6 (neurotransmitter transporter, taurine), member 6	Transporter	Extended
SLC7A8	solute carrier family 7 (cationic amino acid transporter, y+ system), member 8	Transporter	Extended
SLCO1C1	solute carrier organic anion transporter family, member 1C1	Transporter	Extended
SLCO2A1	solute carrier organic anion transporter family, member 2A1	Transporter	Extended
SLCO3A1	solute carrier organic anion transporter family, member 3A1	Transporter	Extended
SLCO4A1	solute carrier organic anion transporter family, member 4A1	Transporter	Extended
SLCO4C1	solute carrier organic anion transporter family, member 4C1	Transporter	Extended
SLCO5A1	solute carrier organic anion transporter family, member 5A1	Transporter	Extended
SLCO6A1	solute carrier organic anion transporter family, member 6A1	Transporter	Extended
SULT1C1	sulfotransferase family, cytosolic, 1C, member 1	Phase II	Extended
SULT1C2	sulfotransferase family, cytosolic, 1C, member 2	Phase II	Extended
SULT1E1	sulfotransferase family 1E, estrogen-preferring, member 1	Phase II	Extended
SULT2A1	sulfotransferase family, cytosolic, 2A, DHEA preferring, member 1	Phase II	Extended
SULT2B1	sulfotransferase family, cytosolic, 2B, member 1	Phase II	Extended
TAP1	transporter 1, ATP-binding cassette, sub-family B (MDR/TAP)	Transporter	Extended
UGT1A10	UDP glucuronosyltransferase 1 family, polypeptide A10	Phase II	Extended
UGT1A4	UDP glucuronosyltransferase 1 family, polypeptide A4	Phase II	Extended
UGT1A5	UDP glucuronosyltransferase 1 family, polypeptide A5	Phase II	Extended
UGT2B10	UDP glucuronosyltransferase 2 family, polypeptide B10	Phase II	Extended
ABCC13	ATP-binding cassette, sub-family C (CFTR/MRP), member 13	Transporter	Extended
ARSA	arylsulfatase A	Modifier	Extended
CAT	catalase	Modifier	Extended
CHST8	carbohydrate (N-acetylgalactosamine 4-0) sulfotransferase 8	Phase II	Extended
CYP19A1	cytochrome P450, family 19, subfamily A, polypeptide 1	Phase I	Extended
CYP26C1	cytochrome P450, family 26, subfamily C, polypeptide 1	Phase I	Extended
CYP27B1	cytochrome P450, family 27, subfamily B, polypeptide 1	Phase I	Extended
CYP2R1	cytochrome P450, family 2, subfamily R, polypeptide 1	Phase I	Extended
CYP2S1	cytochrome P450, family 2, subfamily S, polypeptide 1	Phase I	Extended
CYP46A1	cytochrome P450, family 46, subfamily A, polypeptide 1	Phase I	Extended
CYP4A11	cytochrome P450, family 4, subfamily A, polypeptide 11	Phase I	Extended
CYP4F12	cytochrome P450, family 4, subfamily F, polypeptide 12	Phase I	Extended
CYP4F2	cytochrome P450, family 4, subfamily F, polypeptide 2	Phase I	Extended
CYP4F3	cytochrome P450, family 4, subfamily F, polypeptide 3	Phase I	Extended
CYP4F8	cytochrome P450, family 4, subfamily F, polypeptide 8	Phase I	Extended
CYP4Z1	cytochrome P450, family 4, subfamily Z, polypeptide 1	Phase I	Extended
CYP7B1	cytochrome P450, family 7, subfamily B, polypeptide 1	Phase I	Extended
CYP8B1	cytochrome P450, family 8, subfamily B, polypeptide 1	Phase I	Extended
DHRS13	dehydrogenase/reductase (SDR family) member 13	Phase I	Extended
DHRS2	dehydrogenase/reductase (SDR family) member 2	Phase I	Extended
GPX1	glutathione peroxidase 1	Phase I	Extended
GPX4	glutathione peroxidase 4 (phospholipid hydroperoxidase)	Phase I	Extended
GPX5	glutathione peroxidase 5 (epididymal androgen-related protein)	Phase I	Extended
GPX6	glutathione peroxidase 6 (olfactory)	Phase I	Extended
GSS	glutathione synthetase	Phase I	Extended
GSTCD	glutathione S-transferase, C-terminal domain containing	Phase II	Extended
HNF4A	hepatocyte nuclear factor 4, alpha	Modifier	Extended
HNMT	histamine N-methyltransferase	Phase II	Extended
HSD11B1	hydroxysteroid (17-beta) dehydrogenase 11	Phase I	Extended
HSD17B11	hydroxysteroid (17-beta) dehydrogenase 11	Phase I	Extended
HSD17B14	hydroxysteroid (17-beta) dehydrogenase 14	Phase I	Extended
LOC731356	similar to dehydrogenase/reductase (SDR family) member 4 like 2	Phase I	Extended

MGST1	microsomal glutathione S-transferase 1	Phase II	Extended
MGST2	microsomal glutathione S-transferase 2	Phase II	Extended
MGST3	microsomal glutathione S-transferase 3	Phase II	Extended
MPO	myeloperoxidase	Modifier	Extended
NOS1	nitric oxide synthase 1 (neuronal)	Phase I	Extended
NOS2A	nitric oxide synthase 2A (inducible, hepatocytes)	Phase I	Extended
NOS3	nitric oxide synthase 3 (endothelial cell)	Phase I	Extended
PPARA	peroxisome proliferator-activated receptor alpha	Modifier	Extended
SERPINA7	serpin peptidase inhibitor, clade A (alpha-1 antiproteinase, antitrypsin), member 7	Modifier	Extended
SLC7A7	solute carrier family 7 (cationic amino acid transporter, y+ system), member 7	Transporter	Extended
SOD1	superoxide dismutase 1, soluble (amyotrophic lateral sclerosis 1 (adult))	Modifier	Extended
SOD2	superoxide dismutase 2, mitochondrial	Modifier	Extended
SOD3	superoxide dismutase 3, extracellular precursor	Modifier	Extended
SULF1	sulfatase 1	Phase I	Extended
SULT4A1	sulfotransferase family 4A, member 1	Phase II	Extended
TAP2	transporter 2, ATP-binding cassette, sub-family B (MDR/TAP)	Transporter	Extended
UGT8	UDP glycosyltransferase 8 (UDP-galactose ceramide galactosyltransferase)	Phase II	Extended
XDH	xanthine dehydrogenase	Phase I	Extended
ADHFE1	alcohol dehydrogenase, iron containing, 1	Phase I	Extended
CHST1	carbohydrate (keratan sulfate Gal-6) sulfotransferase 1	Phase II	Extended
CHST10	carbohydrate sulfotransferase 10	Phase II	Extended
CHST11	carbohydrate (chondroitin 4) sulfotransferase 11	Phase II	Extended
CHST12	carbohydrate (chondroitin 4) sulfotransferase 12	Phase II	Extended
CHST13	carbohydrate (chondroitin 4) sulfotransferase 13	Phase II	Extended
CHST2	carbohydrate (N-acetylglucosamine-6-O) sulfotransferase 2	Phase II	Extended
CHST3	carbohydrate (chondroitin 6) sulfotransferase 3	Phase II	Extended
CHST4	carbohydrate (N-acetylglucosamine 6-O) sulfotransferase 4	Phase II	Extended
CHST5	carbohydrate (N-acetylglucosamine 6-O) sulfotransferase 5	Phase II	Extended
CHST6	carbohydrate (N-acetylglucosamine 6-O) sulfotransferase 6	Phase II	Extended
CHST7	carbohydrate (N-acetylglucosamine 6-O) sulfotransferase 7	Phase II	Extended
CHST9	carbohydrate (N-acetylgalactosamine 4-0) sulfotransferase 9	Phase II	Extended
CYP2D7P1	cytochrome P450, family 2, subfamily D, polypeptide 7 pseudogene 1	Phase I	Extended
DDO	D-aspartate oxidase	Phase I	Extended
DHRS1	dehydrogenase/reductase (SDR family) member 1	Phase I	Extended
DHRS12	dehydrogenase/reductase (SDR family) member 12	Phase I	Extended
DHRS3	dehydrogenase/reductase (SDR family) member 3	Phase I	Extended
DHRS4	dehydrogenase/reductase (SDR family) member 4	Phase I	Extended
DHRS4L1	dehydrogenase/reductase (SDR family) member 4 like 1	Phase I	Extended
DHRS4L2	dehydrogenase/reductase (SDR family) member 4 like 2	Phase I	Extended
DHRS7	dehydrogenase/reductase (SDR family) member 7	Phase I	Extended
DHRS7B	dehydrogenase/reductase (SDR family) member 7B	Phase I	Extended
DHRS7C	dehydrogenase/reductase (SDR family) member 7C	Phase I	Extended
DHRS9	dehydrogenase/reductase (SDR family) member 9	Phase I	Extended
DHRSX	dehydrogenase/reductase (SDR family) X-linked	Phase I	Extended
DPEP1	dipeptidase 1 (renal)	Phase I	Extended
FMO6P	flavin containing monooxygenase 6	Phase I	Extended
HAGH	hydroxyacylglutathione hydrolase	Phase I	Extended
IAPP	islet amyloid polypeptide	Modifier	Extended
KCNJ11	potassium inwardly-rectifying channel, subfamily J, member 11	Modifier	Extended
LOC728667	similar to dehydrogenase/reductase (SDR family) member 2 isoform 1	Phase I	Extended
LOC731931	similar to dehydrogenase/reductase (SDR family) member 2 isoform 1	Phase I	Extended
MAT1A	methionine adenosyltransferase I, alpha	Modifier	Extended
METAP1	methionyl aminopeptidase 1	Phase I	Extended
PDE3A	phosphodiesterase 3A, cGMP-inhibited	Phase I	Extended
PDE3B	phosphodiesterase 3B, cGMP-inhibited	Phase I	Extended
PLGLB1	plasminogen-like B1	Phase I	Extended
ATP7A	ATPase, Cu++ transporting, alpha polypeptide (Menkes syndrome)	Modifier	Extended
ATP7B	ATPase, Cu++ transporting, beta polypeptide	Modifier	Extended
CFTR	cystic fibrosis transmembrane conductance regulator	Modifier	Extended

List of drug target genes (based on www.broadinstitute.org/cancer/cga/target):

Gene	Examples of Therapeutic Agents
ABL1	Imatinib, Dasatinib, Nilotinib, ABL1 inhibitors
AKT1	AKT/MTOR inhibitors
AKT2	AKT/MTOR inhibitors
AKT3	AKT/MTOR inhibitors
ALK	Crizotinib, ALK inhibitors
APC	WNT inhibitors
AR	Androgen Deprivation, Enzalutamide
ARAF	Sorafenib, Vemurafenib, Dabrafenib, RAF inhibitors
ASXL1	
ATM	PARP Inhibitors
ATR	PARP Inhibitors
AURKA	AURKA Inhibitors
BAP1	HDAC Inhibitors
BCL2	BCL2 inhibitors
BRAF	Vemurafenib, Dabrafenib, RAF inhibitors, MEK inhibitors
BRCA1	PARP Inhibitor
BRCA2	PARP Inhibitor
BRD2	HDAC Inhibitors, Bromodomain inhibitors
BRD3	HDAC Inhibitors, Bromodomain inhibitors
BRD4	HDAC Inhibitors, Bromodomain inhibitors
c15orf55	HDAC Inhibitors, Bromodomain inhibitors
CCND1	Hormone therapy, CDK4/6 inhibitors
CCND2	CDK4/6 inhibitors,
CCND3	CDK4/6 inhibitors
CCNE1	CDK2 inhibitor
CDH1	
CDK12	PARP inhibitors, Platinum chemotherapy
CDK4	CDK4/6 inhibitors
CDK6	CDK4/6 inhibitors
CDKN1A	CDK inhibitors
CDKN1B	CDK inhibitors
CDKN2A	CDK4/6 inhibitors
CDKN2B	CDK4/6 inhibitors
CEBPA	Transplant
CREBBP	
CRKL	Gefitinib, Erlotinib, EGFR inhibitors, Vemurafenib, Dabrafenib, RAF inhibitors, Dasatinib, SRC inhibitors
CTNNB1	WNT inhibitors
DDR2	Dasatinib
DNMT3A	DNAMT inhibitors
EGFR	Erlotinib, Gefitinib, EGFR Inhibitors
EPHA3	Dasatinib, Ephrin inhibitors
ERBB2	Trastuzumab, Lapatinib, TDM1, Pertuzumab
ERBB3	Pertuzumab
ERBB4	Lapatinib
ERCC2	Cisplatin
ERG	PARP Inhibitor
ERRFI1	Erlotinib, Gefitinib, EGFR Inhibitors
ESR1	Hormonal therapy
ETV1	
ETV4	
ETV5	
ETV6	
EWSR1	
EZH2	EZH2 inhibitors
FBXW7	MTOR inhibitors, Tubulins
FGFR1	FGFR Inhibitors

FGFR2	FGFR Inhibitors
FGFR3	FGFR Inhibitors
FLCN	Everolimus, Temsirolimus, MTOR inhibitors
FLT3	Sunitinib, FLT3 inhibitors
GNA11	MAPK pathway inhibitors
GNAQ	MAPK pathway inhibitors
GNAS	JAK inhibitors
HRAS	MAPK pathway inhibitors
IDH1	IDH inhibitors
IDH2	IDH inhibitors
IGF1R	IFG1-R Inhibitor
JAK2	ruxolitinib, JAK inhibitors
JAK3	tofacitinib, JAK inhibitors
KDR	KDR inhibitors
KIT	Imatinib, Sunitinib, Novel KIT Inhibitors
KRAS	Cetuximab, MEK inhibitors
MAP2K1	Vemurafenib, MEK inhibitors
MAP2K2	MEK inhibitors
MAP2K4	
MAP3K1	
MAPK1	Erlotinib, Gefitinib, EGFR Inhibitors
MAPK3	Erlotinib, Gefitinib, EGFR Inhibitors
MCL1	Tubulins
MDM2	Nutlin, MDM2 inhibitors
MDM4	MDM4 inhibitors
MED12	
MEN1	
MET	Gefitinib, Erlotinib, EGFR inhibitors, Crizotinib, MET inhibitors
MITF	Vemurafenib, Dabrafenib, RAF inhibitors
MLH1	
MLL	HDAC Inhibitors
MPL	Ruxolitinib, JAK2 inhibitors
MSH2	
MSH6	
MTOR	Everolimus, Temsirolimus, MTOR inhibitors
MYC	
MYD88	BTK inhibitors
NF1	PI3K/AKT/MTOR inhibitors, RAF inhibitors, MEK inhibitors
NF2	PI3K/AKT/MTOR inhibitors
NFKBIA	
NKX2-1	
NOTCH1	Notch Inhibitors
NOTCH2	Notch Inhibitors
NPM1	Transplant
NRAS	Vemurafenib, Dabrafenib, RAF inhibitors, MEK inhibitors
NTRK3	PI3K/AKT/MTOR inhibitors, Dasatinib. src inhibitors, IGF1-R inhibitors
PDGFRA	Imatininb
PDGFRB	Imatininb
PIK3CA	PI3K/AKT/MTOR inhibitors
PIK3CB	PI3K/AKT/MTOR inhibitors
PIK3R1	PI3K/AKT/MTOR inhibitors
PTCH1	Vismodegib, hedgehog inhibitors
PTEN	PI3K/AKT/MTOR inhibitors, PARP inhinitors
RAB35	WNT inhibitors
RAF1	Sorafenib, RAF inhibitors
RARA	ATRA, Arsenic
RB1	CDK inhibitors
RET	Sorafenib, vandetinib, RET Inhibitors
RHEB	MTOR inhibitors
RNF43	Porcupine inhibitors
ROS1	Crizotinib
RSPO2	WNT inhibitors

RUNX1	
SMAD2	
SMAD4	
SMARCA4	HDAC
SMARCB1	CDK inhibitors, Vismodegib, Hedgehog inhibitors, HDAC inhibitors
SMO	Vismodegib, hedgehog inhibitors
STK11	Everlolims, Temsirolimus, MTOR inhibitors, Dasatinib, src inhibitors, FAK inhibitors, Phenformin
SYK	SYK inhibitors
TET2	
TMPRSS2	PARP Inhibitors
TP53	Wee1 inhibitors, Chk1 inhibitors, kevetrin, APR-246, nutlins, gene therapy
TSC1	Everolimus, Temsirolimus, MTOR inhibitors
TSC2	Everolimus, Temsirolimus, MTOR inhibitors
VHL	
WT1	
XPO1	SINE agents
ZNRF3	Porcupine inhibitors

Supplementary Table S5: Correlation analyses of DNA methylation and gene expression

A: ADME genes

Cg-probe (450K)	Chromosome	Assigned Gene (UCSC)	RefGene Group (UCSC)	Relation to CpG island	Spearman`s correlation coefficient
cg01054938	chr17	ABCC3	Body	S_Shore	-0.50
cg25928474	chr17	ABCC3	Body	S_Shelf	-0.49
cg02241241	chr21	ABCG1	5'UTR;Body;TSS1500;TSS200	Island	-0.43
cg21410080	chr21	ABCG1	5'UTR;Body;TSS200	Island	-0.52
cg27243685	chr21	ABCG1	5'UTR;Body	S_Shelf	-0.44
cg02370100	chr21	ABCG1	Body	Island	-0.55
cg07397296	chr21	ABCG1	Body	Island	-0.58
cg00222799	chr21	ABCG1	Body	Island	-0.45
cg02396253	chr6	ALDH8A1	1stExon	OpenSea	-0.71
cg20352402	chr6	ALDH8A1	TSS200	OpenSea	-0.71
cg06370069	chr10	CHST3	5'UTR	S_Shelf	-0.49
cg09686390	chr10	CHST3	5'UTR	OpenSea	-0.64
cg24990212	chr8	CYP7B1	Body	N_Shore	-0.66
cg15160198	chr8	CYP7B1	TSS200	Island	-0.55
cg03535659	chr8	CYP7B1	TSS200	Island	-0.52
cg09975850	chr8	CYP7B1	TSS200	Island	-0.55
cg19424531	chr8	CYP7B1	TSS200	Island	-0.66
cg00054210	chr8	CYP7B1	TSS1500	Island	-0.66
cg01510388	chr8	CYP7B1	TSS1500	Island	-0.68
cg13081014	chr1	FMO1	Body	OpenSea	-0.64
cg22005145	chr5	GPX3	TSS1500	N_Shore	-0.55
cg18849169	chr5	GPX3	TSS1500	N_Shore	-0.60
cg08891071	chr5	GPX3	TSS200	N_Shore	-0.42
cg17820459	chr5	GPX3	Body	S_Shore	-0.46
cg12684668	chr5	GPX3	Body	S_Shelf	-0.55
cg18087326	chr1	GPX7	TSS1500	N_Shore	-0.73
cg12640469	chr1	GPX7	TSS1500	N_Shore	-0.59
cg26251270	chr1	GPX7	TSS1500	N_Shore	-0.69
cg22129364	chr1	GPX7	TSS200	Island	-0.44
cg11953272	chr1	GPX7	TSS200	Island	-0.73
cg20950465	chr1	GPX7	1stExon	Island	-0.47
cg16557944	chr1	GPX7	1stExon	Island	-0.50
cg23272399	chr1	GPX7	Body	Island	-0.61
cg02453146	chr1	GPX7	Body	Island	-0.64
cg09161043	chr1	GPX7	Body	S_Shore	-0.48
cg01238044	chr22	GSTT1	Body	N_Shore	-0.76
cg11478607	chr22	GSTT1	TSS200	Island	-0.52
cg02906939	chr2	HNMT	TSS1500	OpenSea	-0.70
cg15441973	chr2	HNMT	TSS1500	OpenSea	-0.72
cg07757007	chr2	HNMT	Body	OpenSea	-0.63
cg07601258	chr6	SLC22A2	Body	N_Shore	-0.65
cg02490934	chr6	SLC22A2	Body	N_Shore	-0.69
cg13717233	chr6	SLC22A2	1stExon	Island	-0.66
cg19561774	chr6	SLC22A2	1stExon	Island	-0.69
cg19627213	chr6	SLC22A2	TSS200	S_Shore	-0.73
cg21755969	chr6	SLC22A2	TSS200	S_Shore	-0.77
cg04294894	chr6	SLC22A2	TSS200	S_Shore	-0.73
cg02666489	chr6	SLC22A2	TSS200	S_Shore	-0.73
cg25545503	chr6	SLC22A2	TSS1500	S_Shore	-0.69
cg07026448	chr6	SLC22A2	TSS1500	S_Shore	-0.66
cg12302621	chr15	SLC28A1	TSS1500	OpenSea	-0.71
cg08271842	chr15	SLC28A1	TSS1500	OpenSea	-0.79

cg10680235	chr15	SLC28A1	5'UTR	OpenSea	-0.73
cg22776451	chr15	SLC28A1	5'UTR	OpenSea	-0.66
cg22294181	chr15	SLC28A1	Body	OpenSea	-0.66
cg03679305	chr1	SLC2A5	Body	N_Shelf	-0.47
cg12763828	chr1	SLC2A5	1stExon;5'UTR;Body	N_Shore	-0.61
cg00310940	chr1	SLC2A5	1stExon;5'UTR;Body	N_Shore	-0.61
cg07787240	chr1	SLC2A5	Body;TSS1500	N_Shore	-0.57
cg07438246	chr3	SLC6A6	5'UTR	S_Shelf	-0.54
cg15527515	chr14	SLC7A8	Body	OpenSea	-0.66
cg02496728	chr3	SLCO2A1	Body	Island	-0.43
cg07780818	chr3	SLCO2A1	TSS1500	S_Shore	-0.53
cg05430989	chr3	SLCO2A1	TSS1500	S_Shore	-0.63
cg06346099	chr6	SOD2	3'UTR	OpenSea	-0.61
cg07073960	chr8	SULF1	TSS200	OpenSea	-0.39

B: Drug target genes

Cg-probe (450K)	Chromosome	Assigned Gene (UCSC)	RefGene Group (UCSC)	Relation to CpG island	Spearman`s correlation coefficient
cg25454116	chr12	CCND2	TSS1500	Island	-0.38
cg26864834	chr12	CCND2	TSS1500	N_Shore	-0.61
cg21057429	chr12	CCND2	TSS1500	N_Shore	-0.56
cg12382902	chr12	CCND2	Body	Island	-0.43
cg18584387	chr12	CCND2	Body	S_Shore	-0.54
cg17655614	chr16	CDH1	TSS1500	N_Shore	-0.55
cg11667754	chr16	CDH1	TSS1500	N_Shore	-0.49
cg01857829	chr16	CDH1	Body	Island	-0.55
cg20716119	chr16	CDH1	Body	Island	-0.67
cg24765079	chr16	CDH1	Body	S_Shore	-0.63
cg09406989	chr16	CDH1	Body	S_Shore	-0.62
cg25156198	chr7	CDK6	Body	OpenSea	-0.42
cg11998200	chr7	CDK6	Body	OpenSea	-0.52
cg19103429	chr7	CDK6	Body	N_Shore	-0.56
cg17217691	chr1	DDR2	TSS1500	OpenSea	-0.56
cg23028772	chr1	DDR2	TSS1500	OpenSea	-0.58
cg21880888	chr1	DDR2	5'UTR	OpenSea	-0.40
cg22740835	chr1	DDR2	5'UTR	OpenSea	-0.52
cg21539842	chr1	DDR2	5'UTR	OpenSea	-0.42
cg04794420	chr12	ERBB3	Body	Island	-0.74
cg10869879	chr12	ERBB3	Body	S_Shore	-0.56
cg00907267	chr12	ERBB3	Body	S_Shore	-0.69
cg11835619	chr12	ERBB3	Body	S_Shore	-0.71
cg06032349	chr21	ERG	5'UTR	Island	-0.67
cg01340163	chr4	KDR	Body	N_Shore	-0.54
cg10740902	chr4	KDR	1stExon;5'UTR	Island	-0.42
cg07893544	chr4	KDR	TSS200	Island	-0.45
cg21161891	chr4	KDR	TSS200	Island	-0.43
cg18177533	chr4	KDR	TSS200	Island	-0.46
cg25613180	chr5	PDGFRB	Body	S_Shore	-0.40
cg14051336	chr5	PDGFRB	Body	S_Shelf	-0.47
cg04173992	chr5	PDGFRB	5'UTR	OpenSea	-0.64
cg11042320	chr5	PDGFRB	5'UTR	OpenSea	-0.47

Supplementary Methods

Study cohort

Metastases (n=20) of ccRCC patients as well as primary ccRCC tissue samples (n=34) of patients, obtained from the Department of Urology and the Department of Pathology (University Hospital Tuebingen, Germany) as well as the Department of Pathology (Robert-Bosch-Hospital, Stuttgart, Germany), were included in the present study. In addition, primary ccRCC tissue from one individual was included as quality control sample to prepare technical replicates for DNA methylation arrays. The study comprised only ccRCC patients of Caucasian origin, who underwent partial or radical nephrectomy or metastases resection between 1996 and 2014, and for which surgical specimens of resected fresh-frozen ccRCC or metastasis tissues were available. Patients' characteristics, clinicopathological features, survival data as well as details about metastases are given in Table 1. In addition, lymph node metastases and local recurrences within the nephrectomy bed were included (Table 1). For five patients, primary tumor and corresponding metastasis samples were available, one patient had one primary tumor and two metastases. In addition, two patients with two metastases each were included. For OCT2 investigations we included selected non-tumor renal tissues of primary ccRCC as described in Fisel et al. [1]. Informed written consent was provided and the use of the tissue was approved by the ethics committee of the University of Tuebingen, Germany.

Study cohort of The Cancer Genome Atlas (TCGA)

Open-access clinical and DNA methylation data of The Cancer Genome Atlas (TCGA) from three independent cohorts of renal cell carcinoma patients including primary ccRCC (KIRC, n=319), chromophobe RCC (KICH, n=66), and papillary RCC (KIRP, n=226) were investigated for whom Illumina Infinium 450K DNA methylation data were available at the time of our analyses.

DNA methylation data of The Cancer Genome Atlas (TCGA) renal cell carcinoma (RCC) cohorts (KIRC, KIRP, KICH)

The following genome-wide DNA methylation data sets from The Cancer Genome Atlas (TCGA; http://cancergenome.nih.gov/) were downloaded on 2014-08-29 via the Cancer Genomics Browser (UC Santa Cruz; https://genome-cancer.soe.ucsc.edu/proj/site/hgHeatmap) and further processed:

The directories TCGA_KIRC_hMethyl450-2014-08-22, TCGA_KIRP_hMethyl450-2014-08-22, and TCGA_KICH_hMethyl450-2014-08-22 contain shifted methylation data from Illumina Infinium 450K Bead chips for the three currently available TCGA RCC cohorts: KIRC (clear cell RCC), KICH (chromophobe RCC), and KIRP (papillary RCC). Thereof, β-values were obtained by adding an offset of 0.5 (cf. https://genome-cancer.soe.ucsc.edu/proj/site/hgHeatmap). The directories incorporate methylation data on 319 (KIRC), 66 (KICH), and 226 (KIRP) primary tumors, respectively. In order to fit the data distributions in the three cohorts to one another, we first computed the probe-wise medians of the β-values over all primary tumors in each RCC entity. As a second step, we performed quantile normalization on the three median profiles (MP) obtained. For each cohort, the individual tumor samples were then adapted to their respective normalized median profile (nMP). In detail, we computed a scaling factor for each β-value by applying linear interpolation based on the respective MP-values (i.e., same RCC entity) and nMP/MP-ratio (in case of ties, mean ratios were calculated). For β-values outside the range of their MP-values, the ratio corresponding to the minimum or maximum MP-value, as appropriate, was chosen. Finally, each β-value was multiplied with its corresponding scaling factor. In addition, based on these data, normalized M-values were computed according to $M = \log2(\beta/(1- \beta))$.

All calculations mentioned above were performed with statistical software R-3.1.1 ([4]) and additional package limma_3.20.9 ([5], [6]).

DNA methylation data of RCC cell lines (n=5) as well as primary ccRCC (n=34) and metastasis (n=20) of our own study cohort: Quality control, data preprocessing, and batch effect correction

Illumina Infinium 450k .idat-files were generated by Service XS (Service XS, Leiden, The Netherlands) using Illumina's GenomeStudio v2011.1 software with Methylation Module v1.9.0, with the default settings as advised by Illumina. No normalization was performed in this step. These .idat-files were imported into statistical software R-3.1.1 using additional package methylumi-2.10.0 ([7]). In addition to the workflow of Service XS, quality control was performed using diagnostic plots and functions of methylumi and lumi_2.16.0 ([8]). In total, 62 chips (34 primary ccRCC – where for three ccRCCs two regions were available and measured on different chips; 20 metastases from primary RCC; 5 RCC cell lines) were included in this study. In addition, two chips of technical replicates of one primary ccRCC were analysed.

Data were preprocessed by applying the following steps: (I) background correction using function methylumi.bgcorr with normal-exponential deconvolution and default parameters ([9]), (II) color bias adjustment with function lumiMethyC, and (III) beta-mixture quantile dilation normalization (BMIQ) for correction of probe design bias ([10]; BMIQ version 1.3, https://code.google.com/p/bmiq). Here, BMIQ was computed on β-values, defined as $\beta = m/(u+m+c)$, where u and m denote the (background corrected and color-bias adjusted) unmethylated and methylated signal intensites and $c = 100$ is a small correction term particularly with regard to low overall intensities (cf. [10]). Based on these preprocessed β-values, M-values were computed according to $M = \log2(\beta/(1- \beta))$.

Since a moderate batch effect was observed - corresponding to three different chip manufacturing dates (May 2013, June 2014, and October 2014) - function removeBatchEffect from R-package limma ([5]) was applied to M-values. Here, sample type membership (i.e., primary tumor, metastasis, or cell line) was considered in order not to remove biologically meaningful effects. Batch-corrected β-values were received from the batch-corrected M-values, applying the inverse formula from above, i.e. $\beta = 2^M/(2^M+1)$. Importantly, the batch-corrected β- and M-values were only used for correlation and cluster analyses as well as graphical representation of the methylation data. Otherwise, a batch factor - corresponding to the three chip

manufacturing dates - was directly considered in the respective analysis described below.

Moreover, as for the TCGA methylation data sets (level 3 data, vide https://tcga-data.nci.nih.gov), β- or M-values for probes with a non-detection probability > 5% were considered as missing. Probes on X- and Y-chromosomes as well as control probes and the 65 SNP probes on the Illumina Infinium 450K Bead chip were excluded. All analyses were based on the remaining 473,864 cg-probes.

Correlation and cluster analyses of genome-wide DNA methylation data

As described above, we used our batch-corrected data (β- or M-values as appropriate, see below) for all cluster analyses.

a. Correlation analysis without TCGA data

Based on the β-values of the 473,864 probes, Spearman's correlation coefficients were computed between samples of the same patient as well as the two regions of the same tumor, as appropriate.

b. Clustering without TCGA data

Hierarchical clustering using Ward's minimum variance method [11, 12] and Manhattan distance was applied to group our chips (including as well as excluding the 5 RCC cell lines).

c. Cluster analysis including the three TCGA RCC cohorts

In order to be able to distinguish between different RCC entities, we first selected probes showing significant and relevant differences between the three TCGA RCC tumor types. Consequently, all probes were chosen which satisfied both, Benjamini-Hochberg adjusted [13] Wilcoxon-Mann-Whitney *P*-value ≤ 5% and absolute difference in median β-values ≥ 20%, in at least one of the pairwise comparisons between the three groups of primary TCGA RCC tumors (KIRC, KIRP, and KICH). For the selected 41,322 probes, we then combined the normalized M-values of the three TCGA RCC tumor types with the batch-corrected M-values of our ccRCC and metastases samples (i.e. excluding the 5 RCC cell lines). M-values were centered

and standardized per sample in order to fit the TCGA data and our data to one another.

R-library ConsenusClusterPlus_1.18.0 ([14],[15]) was then applied for clustering of samples based on the k-means algorithm with Euclidean distance, 1,000 resampling steps, and a maximal cluster number of five. Complete hierarchical clustering with Euclidean distance was used to group cg-probes.

Dendrograms and heatmaps (on batch-corrected β-values) were plotted with R-library gplots_2.14.2 [16].

Determination of differentially methylated cg-sites and of differentially methylated regions (DMR)

As described above, for all further analyses, we (only) used our normalized M-values without batch correction, and rather directly incorporated a factor for the respective array batch in our models. In addition, for each of the three ccRCCs for which two regions per tumor were measured, M-values were averaged over both regions previous to all investigations described below.

Beside the investigations of the 473,864 cg-probes, we performed additional subanalyses excluding probes (I) found by [17] to be cross-reactive with areas of the genome not at the site of interest and/or (II) annotated by R-library Illumina450ProbeVariants.db_1.0.0 to have variants within 50 base pairs of the probed CG with a frequency >1% in the European population (EUR) of the 1000 Genomes Project ([18], [19]). 348,721 of the 473,864 cg-probes remained after consideration of SNPs and cross-reactivity.

a. Differentially methylated cg-sites

For each of the 473,864 or 348,721 cg-probes, linear mixed modeling with R-package limma ([5],[6],[20],[21]) was used to compare between

(I) primary RCCs (n=34) and cell lines (n=5),

(II) metastases (n=20) and cell lines (n=5),

(III) primary RCCs (n=34) and metastases (n=20),

(IV) synchronous (n=7) and metachronous metastases (n=13),

(V) lymph node (n=7) and other metastases (n=13), as well as

(VI) metastases (n=20) and primary RCCs, which were non-metastatic at time of surgery (M0, N0; n=20),

(VII) metastases (n=20) and primary RCCs, for which no metastasis occurred during follow-up (n=13),

(VIII) metastases (n=20) and primary RCCs, for which one or more metastases occurred during follow-up (n=21) as well as

(IX) primary RCCs for which no metastasis occurred (n=13) and for which one or more metastases occurred during follow-up (n=21).

Here, all models included the respective array batch as fixed effect. Additional fixed effects in limma analyses (III) – (IX) were patient's gender and age at surgery of RCC or metastasis. Moreover, in the models applied in (III) – (IX), we incorporated subject as a random effect since for eight patients, pairs or triplets of samples were present (five patients had one RCC and one metastasis each, one patient had one RCC and two metastases, and two patients had two metastases each).

The Benjamini-Hochberg procedure [13] controlling the false discovery rate (FDR) was applied for multiple testing adjustment. R-library qqman_3.1.2 [22] was used for Manhattan plots of the unadjusted *P*-values. Violin plots showing the absolute differences between median β-values (batch-corrected) of ccRCCs and metastases across various genomic features were created with R-library vioplot_0.2 [23].

b. Differentially methylated regions (DMRs)

Based on the results from limma analyses (I) and (III) in a., we performed a search for differentially methylated regions with R-library ChAMP_1.2.8 ([24]), using function champ.lasso. For the comparison of primary RCCs and cell lines, we identified 7155 and 4732 significant DMRs in 473,864 and 348,721 cg-probes, respectively, using default parameters. However, no DMRs were found with these settings for the comparison of primary RCCs and metastases (neither with 473,864 nor with 348,721 cg-probes). We therefore repeated this analysis, requiring a DMR only to contain at least two probes with (I) Benjamin-Hochberg adjusted *P*-value in the respective limma analysis below a relaxed threshold of 15% and (II) positions within a maximum

lasso size of 2,000 base pairs. Results of DMR analyses are given in Supplementary Table S3.

mRNA expression of ADME and drug target genes in our cohort and their correlation to DNA methylation

In addition to the genome-wide DNA methylation data, we measured mRNA expression for each sample in our cohort by the Affymetrix Human Transcriptome Array 2.0 (HTA 2.0). For the 59 HTA 2.0 arrays (34 primary ccRCC, 20 metastases from primary RCC, 5 RCC cell lines), quality control and preprocessing was performed with Affymetrix Expression Console (Build 1.4.1.46) and R-3.1.1. Due to low quality, four arrays (three ccRCCs, one pancreas metastasis; none corresponding to patients with several samples in our cohort) were excluded from further analyses.

In our first preprocessing step, Robust Multi-array Average [25] was applied separately to each of the three sample types (ccRCC (n=31), metastasis (n=19), RCC cell line (n=5); Expression Console). Based on the resulting log2 signal intensities, we then performed a median normalization between the sample types in R-3.1.1. To be more precise, probe sets not annotated to chromosomes 1-22 were excluded and the median log2 signal intensity of each array on the remaining probe sets was computed. For each of the three sample types, we then calculated the median of these medians (MOM). For each array, median normalized log2 signals were then obtained by subtracting the MOM of the corresponding sample type and adding the MOM of the ccRCCs.

Based on these data, HTA 2.0 probe sets significantly differentially expressed between ccRCC (n=31) and RCC cell lines (n=5) were identified by R-package limma ([5],[6],[20]), thereby correcting for three different array batches.

A list of 298 ADME related genes, which is based on the PharmaADME initiative (www.pharmaadme.org), as well as non-overlapping 135 drug targets (based on www.broadinstitute.org/cancer/cga/target) was assembled (see Supplementary Table S4). 20 of the ADME genes (*ABCB7, ATP7A, CHST7, DHRS4, DHRSX, LOC728667, LOC731356, LOC731931, SERPINA7, SULT1A3, SULT1C1, UGT1A1,*

UGT1A10, UGT1A3, UGT1A4, UGT1A6, UGT1A7, UGT1A8, UGT1A9, UGT2A1) and 2 drug targets (*AR, ARAF*) were not annotated on the HTA 2.0 array. Concerning the Illumina Infinium 450K Bead chip, no cg-probes were annotated to 9 ADME genes (*ABCB7, ATP7A, CHST7, DHRSX, LOC728667, LOC731356, LOC731931, SERPINA7, SULT1C1*) and 2 drug targets (*AR, ARAF*). In total, for 278 ADME genes (95.9%) and 133 drug targets (98.5%), at least one probe was annotated on the HTA 2.0 array and the Illumina Infinium 450K Bead chip.

Next, we selected those HTA 2.0 probe sets which were annotated to an ADME or drug target gene and showed a significant and relevant expression difference (absolute log2 fold change>1, mean log2 signal intensity>6 in at least one of both groups) between primary tumors and cell lines in the limma analysis described above.

Similarly, we chose those cg-sites which were annotated to an ADME or drug target gene and differed significantly between primary tumors and cell lines in the limma analysis (I) of the methylation data described above and showed a relevant difference in the batch-corrected β-values (absolute difference in medians >10%). For 38 of the 278 ADME genes and 14 of the 133 drug target genes, at least one such cg-site as well as at least one such probe was detected. For each of these genes, we calculated Spearman's correlation coefficients between the log2 signals of the selected HTA 2.0 probe sets and the batch-corrected β-values of the selected cg-sites annotated to the considered gene, over the 31 ccRCCs and 5 RCC cell lines. Supplementary Table S5 shows the significantly negatively correlated probe sets and cg-sites for the ADME and drug target genes, thereby excluding cross-reactive cg-probes and cg-probes annotated to have non-rare SNPs within 50 bp of the probed CpG.

RNA sequencing data of the TCGA ccRCC cohort (KIRC) and correlation to DNA methylation for *SLC22A2/OCT2*

For the TCGA ccRCC cohort (KIRC), RNA sequencing data (Illumina HiSeq 2000 RNA Sequencing platform) were received from the file TCGA_KIRC_exp_HiSeqV2-2015-02-24, downloaded on 2015-10-20 via the Cancer Genomics Browser. The file contained log2(x+1)-transformed data for 533 primary tumors on 20,530 genes. Here,

x are RSEM (RNA-Seq by Expectation Maximization) expression estimates, which were normalized to set the upper quartile count at 1000 (for details, vide http://cancergenome.nih.gov/ and https://genome-cancer.soe.ucsc.edu/proj/site/hgHeatmap).

For the TCGA ccRCC cohort, both RNA sequencing data and 450k methylation data (vide above) was available for 318 primary tumors. For those, Spearman's correlation coefficients were computed between *SLC22A2/OCT2* expression data and β-values of 21 cg-probes annotated to *SLC22A2/OCT2*.

DNA methylation and mRNA expression of *SLC22A2/OCT2* in our study cohort

For quantitative DNA methylation analyses of *SLC22A2* in our study cohort, matrix-assisted laser desorption ionization time-of-flight mass spectrometry (MALDI-TOF MS) was applied as previously described [1; 26]. Mass spectra were obtained using the MassARRAY compact system and evaluated using the EpiTYPER 1.0 software. mRNA levels of *SLC22A2/OCT2* were quantified using TaqMan technology and normalized to β-actin levels as previously described [1].

OCT2 protein expression in our study cohort

Tissue microarray sections containing metastases, primary tumor samples and non-tumor tissues were processed and immunostained as previously described [1]. For immunostaining a previously validated antibody against OCT2 was used [26]. The slides were scanned using a digital Mirax scanner (Carl Zeiss, MicroImaging, Jena, Germany). For the evaluation of immunohistochemical staining, semiquantitative grading of membranous OCT2 staining was based on a comparison of all scanned TMA dots using an image based software platform (Tissue Studio, Definiens) as previously validated and described [27].

Treatment of Caki-2 cells with 5-Aza-2´-deoxycytidine (decitabine)

To measure effects of DNA methylation exponentially growing cells were treated with 1µM 5-Aza-2'-deoxycytidine (decitabine) (Sigma-Aldrich) for 4 days and total RNA

and DNA was extracted. To determine the effects of cisplatin and 5-Aza-2'-deoxycytidine, cells were pretreated for 72 h with 1µM 5-Aza-2'-deoxycytidine and then treated with 10 µM cisplatin for another 48h. Cells were stained with Annexin V/FITC and propidium iodide and analysed by flow cytometry, as previously described [28].

Quantification of total DNA methylation

5-methyl-2'-deoxycytidine (5-meC) content was determined by LC-MS-MS analysis as previously described [29]. DNA methylation status is given as percentage of 5-methyl-2'-deoxycytidine content relative to total cytosine residues.

mRNA expression levels of 55 SLC transporters

mRNA levels of 55 SLC transporters were quantified using TaqMan technology as previously described [30].

References

[1] Fisel P, Kruck S, Winter S, Bedke J, Hennenlotter J, Nies AT, et al. DNA methylation of the SLC16A3 promoter regulates expression of the human lactate transporter MCT4 in renal cancer with consequences for clinical outcome. Clin Cancer Res. 2013;19(18):5170-81.

[2] Hoadley KA, Yau C, Wolf DM, Cherniack AD, Tamborero D, Ng S et al. Multiplatform analysis of 12 cancer types reveals molecular classification within and across tissues of origin. Cell. 2014;158:929-944

[3] The Cancer Genome Atlas Research Network et al. The Cancer Genome Atlas Pan-Cancer analysis project. Nature Genetics. 2013;45,1113–1120

[4] R Core Team. R: A language and environment for statistical computing. R Foundation for Statistical Computing, Vienna, Austria. 2014. http://www.R-project.org

[5] Smyth GK. Limma: linear models for microarray data. In: 'Bioinformatics and Computational Biology Solutions using R and Bioconductor'. R. Gentleman, V. Carey, S. Dudoit, R. Irizarry, W. Huber (eds), Springer, New York. 2005. pages 397-420.

[6] Smyth GK, Speed TP. Normalization of cDNA microarray data. Methods. 2003;31, 265-273.

[7] Davis S, Du P, Bilke S, Triche T Jr, Bootwalla M (2014). methylumi: Handle Illumina methylation data. R package version 2.10.0.

[8] Du P, Kibbe WA, Lin SM. lumi: a pipeline for processing Illumina microarray. Bioinformatics. 2008;24(13):1547-1548

[9] Triche TJ Jr, Weisenberger DJ, Van Den Berg D, Laird PW, Siegmund KD. Low-level processing of Illumina Infinium DNA Methylation BeadArrays. Nucleic Acids Res.2013;41(7):e90.

[10] Teschendorff AE, Marabita F, Lechner M, Bartlett T, Tegner J, Gomez-Cabrero D, Beck S. A beta-mixture quantile normalization method for correcting probe design bias in Illumina Infinium 450 k DNA methylation data. Bioinformatics. 2013;29(2):189-96.

[11] Murtagh, F and Legendre, P. Ward's hierarchical agglomerative clustering method: which algorithms implement Ward's criterion? Journal of Classification. 2014;31:274-295

[12] Ward JH. Hierarchical Grouping to Optimize an Objective Function. Journal of the American Statistical Association. 1963;58:236-244

[13] Benjamini Y, Hochberg Y. Controlling the false discovery rate: a practical and powerful approach to multiple testing. Journal of the Royal Statistical Society Series B. 1995;*57*, 289-300.

[14] Wilkerson M, Waltman P (2013). ConsensusClusterPlus: ConsensusClusterPlus. R package version 1.18.0.

[15] Monti S, Tamayo P, Mesirov J, Golub T. Consensus Clustering: A Resampling-Based Method for Class Discovery and Visualization of Gene Expression Microarray Data. Machine Learning. 2003; 52, 91-118.

[16] Warnes GR, Bolker B, Bonebakker L, Gentleman R, Huber W, Liaw A, et al. (2014). gplots: Various R programming tools for plotting data. R package version 2.14.2. http://CRAN.R-project.org/package=gplots

[17] Chen YA, Lemire M, Choufani S, Butcher DT, Grafodatskaya D, Zanke BW, et al. Discovery of cross-reactive probes and polymorphic CpGs in the Illumina Infinium HumanMethylation450 microarray. Epigenetics. 2013;11;8(2).

[18] Butcher, L (2013). Illumina450ProbeVariants.db: Annotation Package combining variant data from 1000 Genomes Project for Illumina HumanMethylation450 Bead Chip probes. R package version 1.0.0.

[19] Genomes Project Consortia, Abecasis GR, Auton A, Brooks LD, DePristo M A, Durbin RM, Handsaker RE, Kang HM, Marth GT, McVean GA. An integrated map of genetic variation from 1,092 human genomes. Nature. 2012;491(7422):56-65.

[20] Smyth GK. Linear models and empirical Bayes methods for assessing differential expression in microarray experiments. Statistical Applications in Genetics and Molecular Biology. 2004;Vol. 3, No. 1, Article 3.

[21] Smyth GK, Michaud J, Scott H. The use of within-array replicate spots for assessing differential expression in microarray experiments. Bioinformatics. 2005;21(9), 2067-2075.

[22] Turner S (2014). qqman: Q-Q and manhattan plots for GWAS data. R package version 0.1.2. http://CRAN.R-project.org/package=qqman

[23] Adler D (2005). Vioplot: Violin plot. R package version 0.2. http://wsopuppenkiste.wiso.uni-goettingen.de/~dadler

[24] Morris T, Butcher L, Feber A, Teschendorff A, Chakravarthy A, Beck S. 2014. ChAMP: Chip Analysis Methylation Pipeline for Illumina HumanMethylation450. R package version 1.2.8.

[25] Irizarry RA; Hobbs B; Collin F; Beazer-Barclay YD; Antonellis KJ; Scherf U; Speed TP. Exploration, normalization, and summaries of high density oligonucleotide array probe level data. Biostatistics. 2003;4 (2): 249–64.

[26] Schaeffeler E, Hellerbrand C, Nies AT, Winter S, Kruck S, Hofmann U, van der Kuip H, Zanger UM, Koepsell H, Schwab M. DNA methylation is associated with downregulation of the organic cation transporter OCT1 (SLC22A1) in human hepatocellular carcinoma. Genome Med. 2011;3(12):82.

[27] Fisel P, Stühler V, Bedke J, Winter S, Rausch S, Hennenlotter J, Nies AT, Stenzl A, Scharpf M, Fend F, Kruck S, Schwab M, Schaeffeler E. MCT4 surpasses the prognostic relevance of the ancillary protein CD147 in clear cell renal cell carcinoma. Oncotarget. 2015;6(31):30615-27.

[28] Dengler MA, Weilbacher A, Gutekunst M, Staiger AM, Vohringer MC, Horn H, Ott G, Aulitzky WE, van der Kuip H. Discrepant NOXA (PMAIP1) transcript and NOXA protein levels: a potential Achilles' heel in mantle cell lymphoma. Cell Death Dis. 2014;5:e1013

[29] Hocher B, Haumann H, Rahnenführer J, Reichetzeder C, Kalk P, Pfab T, Tsuprykov O, Winter S, Hofmann U, Li J, Püschel GP, Lang F, Schuppan D, Schwab M, Schaeffeler E. Maternal eNOS Deficiency Determines a Fatty Liver Phenotype of the Offspring in a Sex Dependent Manner. Epigenetics. 2016 May 13:0. [epub ahead of print].

[30] Nies AT, Schaeffeler E, van der Kuip H, Cascorbi I, Bruhn O, Kneba M, Pott C, Hofmann U, Volk C, Hu S, Baker SD, Sparreboom A, Ruth P, Koepsell H, Schwab M. Cellular uptake of imatinib into leukemic cells is independent of human organic cation transporter 1 (OCT1). Clin Cancer Res. 2014;20(4):985-94.
